# Activity of tafasitamab in combination with rituximab in subtypes of aggressive lymphoma

**DOI:** 10.3389/fimmu.2023.1220558

**Published:** 2023-07-31

**Authors:** Maria Patra-Kneuer, Gaomei Chang, Wendan Xu, Christian Augsberger, Michael Grau, Myroslav Zapukhlyak, Kristina Ilieva, Karin Landgraf, Doris Mangelberger-Eberl, Kasra Yousefi, Philipp Berning, Katrin S. Kurz, German Ott, Pavel Klener, Cyrus Khandanpour, Pedro Horna, Jürgen Schanzer, Stefan Steidl, Jan Endell, Christina Heitmüller, Georg Lenz

**Affiliations:** ^1^ Translational Research, MorphoSys AG, Planegg, Germany; ^2^ Department of Medicine A, Hematology, Oncology and Pneumology, University Hospital Münster, Münster, Germany; ^3^ Department of Clinical Pathology, Robert-Bosch-Krankenhaus and Dr. Margarete Fischer-Bosch Institute for Clinical Pharmacology, Stuttgart, Germany; ^4^ Institute of Pathological Physiology, First Faculty of Medicine, Charles University Prague, Prague, Czechia; ^5^ First Medical Department, Department of Hematology, Charles University General Hospital Prague, Prague, Czechia; ^6^ Hematology and Oncology Clinic, University of Lübeck and University Hospital Schleswig-Holstein, Lübeck, Germany; ^7^ Division of Hematopathology, Mayo Clinic, Rochester, MN, United States

**Keywords:** antibody therapy, lymphoma, tumor immunology, CD19, CD20, tafasitamab, rituximab

## Abstract

**Background:**

Despite recent advances in the treatment of aggressive lymphomas, a significant fraction of patients still succumbs to their disease. Thus, novel therapies are urgently needed. As the anti-CD20 antibody rituximab and the CD19-targeting antibody tafasitamab share distinct modes of actions, we investigated if dual-targeting of aggressive lymphoma B-cells by combining rituximab and tafasitamab might increase cytotoxic effects.

**Methods:**

Antibody single and combination efficacy was determined investigating different modes of action including direct cytotoxicity, antibody-dependent cell-mediated cytotoxicity (ADCC) and antibody-dependent cellular phagocytosis (ADCP) in *in vitro* and *in vivo* models of aggressive B-cell lymphoma comprising diffuse large B-cell lymphoma (DLBCL) and Burkitt lymphoma (BL).

**Results:**

Three different sensitivity profiles to antibody monotherapy or combination treatment were observed in *in vitro* models: while 1/11 cell lines was primarily sensitive to tafasitamab and 2/11 to rituximab, the combination resulted in enhanced cell death in 8/11 cell lines in at least one mode of action. Treatment with either antibody or the combination resulted in decreased expression of the oncogenic transcription factor MYC and inhibition of AKT signaling, which mirrored the cell line-specific sensitivities to direct cytotoxicity. At last, the combination resulted in a synergistic survival benefit in a PBMC-humanized Ramos NOD/SCID mouse model.

**Conclusion:**

This study demonstrates that the combination of tafasitamab and rituximab improves efficacy compared to single-agent treatments in models of aggressive B-cell lymphoma *in vitro* and *in vivo*.

## Introduction

Aggressive lymphoma comprises a heterogeneous group of different lymphoid malignancies. While diffuse large B-cell lymphoma (DLBCL) represents the most frequent subtype in adults, other subtypes such as Burkitt lymphoma (BL) are rare (1, 2). DLBCL represents a heterogeneous diagnostic category and various molecular subtypes can be distinguished. Based on gene expression profiling, two major subtypes were initially differentiated: activated B-cell-like DLBCL (ABC DLBCL) and germinal center B-cell-like DLBCL (GCB DLBCL) (3). Additionally, roughly 15% of DLBCL patients cannot be categorized into ABC or GCB DLBCL and are referred as unclassifiable (4, 5). Recent studies using next generation sequencing detected molecular subtypes within and beyond ABC and GCB DLBCL, further improving our understanding of the molecular pathogenesis of DLBCL (6–8).

The monoclonal anti-CD20 antibody rituximab was approved for the treatment of patients with malignant lymphoma by the Food and Drug Administration (FDA) already in 1997 (9). In 2006, rituximab was approved in combination with cyclophosphamide, doxorubicin hydrochloride, vincristine and prednisolone (R-CHOP) for the treatment of patients with newly diagnosed DLBCL and R-CHOP remains the predominant standard of care for this patient population, achieving cure rates of approximately 60-70% (10). However, a substantial proportion of DLBCL patients is refractory or relapses after initial response following R-CHOP, indicating that there is still a high unmet medical need (11, 12). For patients with BL, more intense chemotherapeutic regimens in combination with rituximab are commonly used (13).

Besides CD20, the B-cell surface protein CD19 emerged as an attractive target for antibody-based and cellular therapies during the past years (14, 15). CD19 is a specific B-cell marker, homogeneously expressed throughout B-cell differentiation from the pro-B-cell stage on (14). It has been shown that CD19 and CD20 expression can be heterogeneous in lymphoma subpopulations (16–18) and that CD19 expression is preserved in small CD20-negative tumor subpopulations or after CD20-targeted immunotherapy (17, 19). Therefore CD19- and CD20-targeting antibodies might complement each other in the treatment of patients with B-cell lymphomas, as CD19 might compensate for CD20-low/-negative settings and vice versa.

Tafasitamab is a CD19-targeting, Fc-modified (S239D/I332E mutations) humanized monoclonal antibody immunotherapy mediating antibody-dependent cellular cytotoxicity (ADCC), antibody-dependent cellular phagocytosis (ADCP) and direct cytotoxicity (20, 21). Tafasitamab was granted accelerated approval by the FDA in 2020 and conditional marketing authorization by the EMA in 2021 for the treatment of transplant-ineligible adult patients with relapsed or refractory (R/R) DLBCL in combination with the immunomodulatory drug lenalidomide.

Due to the overlapping modes of action of tafasitamab and rituximab (direct cytotoxicity, ADCC and ADCP), we hypothesized that a combination of these two antibodies could increase tumor cell killing in aggressive lymphoma subtypes compared to the respective single antibodies. Hence, we investigated the combination approach in preclinical *in vitro* and *in vivo* lymphoma models.

## Materials and methods

### Reagents

Tafasitamab was provided by MorphoSys and rituximab was purchased from Roche.

### Cell lines

Human B-cell lymphoma cell lines were obtained from ATCC or DSMZ or kindly provided by Louis Staudt (National Institutes of Health, Bethesda). OCI-Ly1, OCI-Ly2, OCI-Ly3, OCI-Ly4, OCI-Ly7, OCI-Ly10, OCI-Ly19 and TMD8 were cultured in Iscove’s modified Dulbecco medium supplemented with 10% FCS or 20% human plasma. All other cell lines were cultured in RPMI 1640 medium (Gibco) with 10%-20% FCS (Sigma). All cells were cultured at 37°C, 5% CO2 (**Supplementary T** **able S1**).

### Determination of CD19/CD20 expression of primary patient samples and cell lines

Primary patient samples: Immunohistochemistry with antibodies against CD20 (clone L26; Cell Marque, Rocklin, CA, USA) and CD19 (clone MRQ-36; Cell Marque) was performed on tissue microarrays (TMA) containing 2 or 3 cores. TMA blocks were constructed from archival formalin-fixed paraffin-embedded DLBCL and BL specimens, as previously described (22). Stains were performed on a semi-automated immunostainer (Lab Vision 720, Thermo Fisher Scientific (Waltham, Massachusetts, USA). Cases were considered positive if >30% of tumor cells expressed the respective antigen. In most cases, tumor cells were either entirely negative or 100% positive. Histological evaluation of staining was done by two expert hematopathologists.

Flow cytometric analysis was performed on primary biopsy specimens from 32 patients with DLBCL (n=29) or Burkitt lymphoma (n=3). Small pieces of lymph node or tissue were received in ice-cold RPMI medium, grinded through a wire mesh to produce a cell suspension in bovine serum albumin and kept at 4°C for up to 96 hours after collection. The samples were then incubated for 15 minutes at room temperature with a cocktail of 7 monoclonal antibodies (all from BD Biosciences): Kappa-FITC (F0434), Lambda-PE (R0437), CD45-PerCP-Cy 5.5 (340665), CD5-PE-Cy7 (348800), CD10-APC (340922), CD20-APC-H7 (641405), CD19-V450 (560353). Stained cells were resuspended in phosphate buffer saline (PBS) and measured on a FACSCanto II flow cytometer (BD Biosciences). Listmode files were analyzed on Kaluza version 2.1 (Beckman Coulter, Brea, CA). Sequential gating and real time color coding was used to identify immunophenotypically abnormal B-cells consistent with the lymphoma cell population, in addition to background polytypic B-cells and CD5-positive T-cells. Thresholds for antigen positivity were defined for each case, based on the percentile 95 fluorescence intensity of background T-cells (internal negative control). In addition, fluorescence intensities of tumor cells were normalized to background polytypic B-cells (internal positive control) using the median fluorescence ratio (MFR).

Cell lines: The average surface expression of CD19 and CD20 receptors on the cell lines was analyzed using the Quantibrite system (BD Biosciences), according to manufacturer’s instructions. For each individual cell line, mean fluorescence intensity (MFI) values were measured upon staining with PE-labeled anti-CD19 (clone HIB19, Biolegend), and anti-CD20 (clone 2H7, Biolegend) antibodies, and the MFI values of reference bead were used to correlate MFI to the number of antibodies bound per cell via linear regression.

### Isolation of effector cells

Whole blood was collected from healthy volunteers. PBMCs were isolated via density-gradient centrifugation using Biocoll (Biochrome) or Pancoll (PAN Biotech) separating solution and SepMate tubes (STEMCELL Technologies). Monocytes and natural killer (NK) cells were isolated from PBMCs according to the manufacturer’s protocol (Miltenyi Biotec). NK cells were negatively selected using biotin-conjugated antibodies and magnetic MicroBeads by magnetic-activated cell sorting (MACS). Monocytes were positively selected using CD14 MicroBeads by MACS. The isolated cells were re-suspended in RPMI 1640 medium supplemented with 10% FCS and 1x GlutaMax. Monocytes were maturated into macrophages in T-flasks for 6 to 7 days in the presence of 50 ng/mL M-CSF.

### ADCC assay

NK cells were used as effector cells and lymphoma cell lines as target cells. Target cells were stained with 1 µM carboxyfluorescein succinimidyl ester (CFSE) and incubated with the effectors at varying effector: target (E:T) ratios (0.5:1, 1:1, 3:1) with or without antibodies (1 or 10 nM) for 2-2.5 hours at 37°C and 5% CO2. Subsequently, dead cells were stained with 1 µg/ml 4’,6-diamidino-2-phenylindole (DAPI). The assay was read out using flow cytometry. Cell populations were gated for viable (DAPI-negative) and dead target cells (DAPI-positive). The antibody-specific lysis was defined by subtracting the percentage of dead target cells in the control sample (target and NK cells without antibody) from the one in the treated sample (target and NK cells with antibody).

### ADCP assay

After maturation, macrophages were stained with 2 µM CFSE and detached using 5 mM EDTA and scraping. After washing with PBS, macrophages were re-suspended in RPMI 1640 medium supplemented with 10% FCS and 1x GlutaMax and allowed to re-attach in 96-well culture plates for 24 hours. Lymphoma cell lines (target cells) were labelled using CellTrace Violet Cell Proliferation Kit (Invitrogen) and co-cultured with macrophages with or without antibodies (10 nM) for 3 hours at a 2:1 E:T ratio. The cells were detached using 0.05% Trypsin-EDTA, transferred into multi-well plates and the assay was read out using flow cytometry. Phagocytosis was defined as the percentage of CellTrace Violet+CFSE+ cells out of all CellTrace Violet+ cells.

### Cell viability assay

Direct cytotoxicity was analyzed using CellTiter Glo kit according to the manufacturer’s protocol (Promega). Lymphoma cells were incubated with a single dose (5 nM) or different doses of tafasitamab, rituximab or the combination on white 96 flat-well plates with clear bottom for 24 or 96 hours at 37°C and 5% CO2. Reduction of cell viability was defined as the percentage of luminescence reduction compared to medium control sample.

### RNA sequencing and gene expression profiling

Gene expression of SU-DHL-6 cells after 6-, 12-, 18- and 24-hour treatment with tafasitamab, rituximab or their combination was profiled as previously described (23, 24). In brief, 6-, 12-, 18- and 24-hours following treatment with either tafasitamab or rituximab alone or their combination in SU-DHL-6 cells. Library preparation was performed using the NEBNext Ultra II Directional RNA Library Prep Kit (New England Biolabs) and the library was subsequently sequenced on a NovaSeq 6000 (Illumina) according to the manufacturers’ protocol. Gene expression changes were measured in two independent biological replicates for each time point. Sequenced reads of mRNA were aligned against the human transcriptome using HISAT2 (25). Aligned sequence counts were then aggregated for genes using RNA-Seq by expectation maximization (RSEM) (26). Gene expression changes in treated SU-DHL6 cells were compared to untreated cells. A non-negative binomial test (Bioinformatics Toolbox of MATLAB^®^ R2020a, The MathWorks^®^ Inc.) was used to calculate p values of gene regulation comparing tafasitamab and/or rituximab with untreated control over all time points or comparing the combination with each single compound. The Benjamini and Hochberg method was used to correct for multiple hypothesis testing and compute false discovery rates (FDRs) (27). To analyze in an unbiased fashion which biological processes were affected by tafasitamab and/or rituximab, we performed a gene set enrichment analysis (GSEA) (28) as previously described (23, 24), testing an integrated database of previous signatures with respect to the gene ranking by tafasitamab and/or rituximab regulation. This database contained signatures from the Molecular Signatures Database v7.1 (29), the Staudt laboratory library (30), unsupervised SDCM signatures of gene expression heterogeneity in DLBCL (31), and signatures from previous analyses (23). GSEA p -values were computed by permutation tests and FDRs were computed relative to respective signature families.

### Western blotting

Western blotting was performed as previously described (23). In brief, whole cell lysates were harvested from cultured cell lines in Phospho-Safe extraction reagent (EMD Millipore) and protein was quantified using the BCA assay (Thermo Scientific). Lysates were separated by SDS-PAGE on 10-12% polyacrylamide gels and transferred to polyvinylidene difluoride membranes (EMD Millipore). Except of anti-α-Tubulin (clone DM1A, Sigma), phospho-PRAS40 (Thr246) (polyclonal, Thermo Scientific) and anti-c-MYC (clone Y69, Abcam), all other antibodies were purchased from Cell Signaling: anti-phospho-AKT (Ser473) (clone D9E), anti-phospho-AKT (Thr308) (clone C31E5E), anti-phospho-S6 ribosomal protein (Ser235/236) (Cat# 2211) and anti-AKT (Cat# 9272).

### Analysis of MYC expression via flow cytometry

Lymphoma cells were incubated with 5 nM tafasitamab, rituximab or the combination for 24 or 48 hours in 96 flat-well plates at 37°C and 5% CO2. Next, the cells were permeabilized using Cytofix/Cytoperm kit (BD Biosciences), stained with anti-MYC Alexa Fluor 647 (clone D84C12, Cell Signaling) or isotype control (clone DA1E, Cell Signaling) and analyzed by flow cytometry. MFI of treated cells was normalized to untreated control cells.

### *In vivo* xenograft mouse studies

Ramos cells (1×10^6^) and human PBMC (5×10^6^) were mixed and co-engrafted subcutaneously in the flank of female non-obese diabetic/severe combined immunodeficient (NOD/SCID) mice (NOD.CB17-Prkdc^scid^/Rj, Janvier) on day 0. PBMCs isolated from whole blood of a total of three healthy donors were used. Intraperitoneal treatment with vehicle (PBS), tafasitamab (0.3/1 mg/kg), rituximab (0.3/0.6/1 mg/kg) or the combination commenced on day 0. Mice were treated twice weekly for six weeks. Tumor volume was monitored three times weekly. Tumor volume was calculated according to the following formula: 1/2 × (length × width^2^). Animals were sacrificed at a tumor volume ≥ 1.5 cm^3^. In survival analyses, tumor volume of 500 mm^3^ was selected as an endpoint. The animal experimentation was approved by ‘‘Landesamt für Gesundheit und Soziales Berlin’’ (Reg 0010/19).

### Statistical analysis

All statistical analyses were performed with GraphPad Prism software (RRID : SCR_002798), versions 8 or 9. Paired two-tailed t-tests were used for statistical comparisons of *in vitro* data. *In vivo* data was analyzed using Mann-Whitney (tumor volume comparisons) or Mantel-Cox (survival) tests. Correlation analyses were performed using Pearson’s test. *p<0.05, **p<0.01, ***p<0.001, ****p<0.0001, n.s. not significant.

### About tafasitamab

Tafasitamab is a humanized Fc-modified cytolytic CD19 targeting monoclonal antibody. In 2010, MorphoSys licensed exclusive worldwide rights to develop and commercialize tafasitamab from Xencor, Inc. Tafasitamab incorporates an XmAb® engineered Fc domain, which mediates B-cell lysis through apoptosis and immune effector mechanism including Antibody-Dependent Cell-Mediated Cytotoxicity (ADCC) and Antibody-Dependent Cellular Phagocytosis (ADCP). In January 2020, MorphoSys and Incyte entered into a collaboration and licensing agreement to further develop and commercialize tafasitamab globally. Following accelerated approval by the U.S. Food and Drug Administration in July 2020, tafasitamab is being co-commercialized by MorphoSys and Incyte in the United States. Conditional/Accelerated approvals were granted by the European Medicines Agency and other regulatory authorities. Incyte has exclusive commercialization rights outside the United States. XmAb® is a registered trademark of Xencor Inc.

## Results

### CD19 and CD20 expression levels in primary patient samples and cell lines of B-cell lymphoma

**Figure 1:**
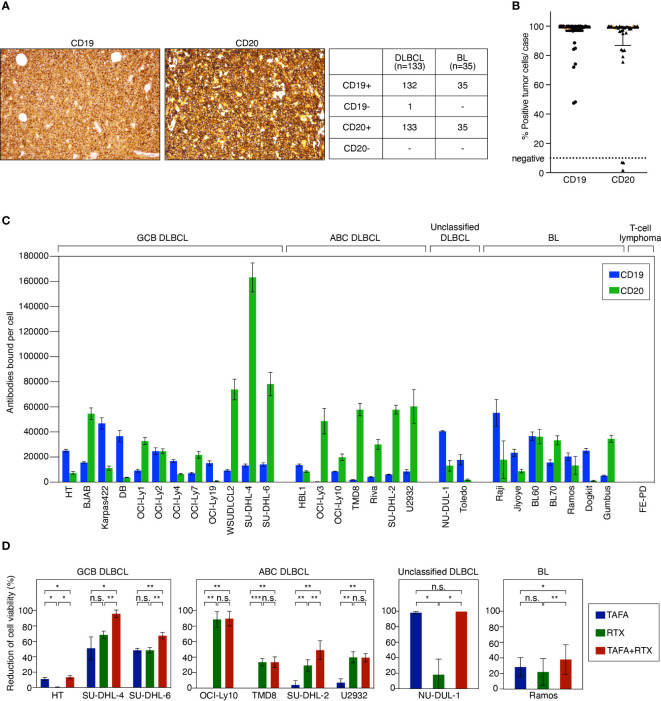
CD19 and CD20 antigen expression levels and impact of tafasitamab, rituximab and the combination of both antibodies on cell viability. **(A)** Representative CD19 and CD20 IHC stainings of lymphoma patient samples and determination of CD19 and CD20 expression in primary DLBCL and BL cases. **(B)** Quantification of CD19 and CD20 expression in patient samples using flow cytometry. 27 DLBCL (black) and 3 BL (orange) cases were analyzed. **(C)** Quantification of CD19 and CD20 surface expression on lymphoma cell lines using flow cytometry. The number of CD19 and CD20 molecules per cell (antibodies bound per cell) was determined with anti-CD19 PE and anti-CD20 PE antibodies and Quantibrite PE beads (BD). Data were shown as mean values with standard deviation (SD) from at least three independent experiments. **(D)** B-cell lymphoma cells were cultured without antibody, with 5 nM tafasitamab (TAFA), 5 nM rituximab (RTX) or the combination of both (TAFA + RTX) for 24 hours (SU-DHL-6) or 96 hours (all other cell lines). Cell viability was determined using the Cell-Titer Glo assay and normalized to untreated cells. The graphs show mean values with SD of 3 to 6 independent experiments. Statistical analysis: paired t test. *p<0.05, **p<0.01, ***p<0.001, n.s., not significant.

To determine the target expression on lymphoma patient samples, 133 DLBCL and 35 BL cases were evaluated by immunohistochemistry (IHC) for CD19 and CD20 expression, respectively. One DLBCL case was found to be negative for CD19 and positive for CD20, while all other cases co-expressed both markers (**Figure 1A**). Quantitative analysis of CD19 and CD20 present on the cell surface was performed using flow cytometry on primary biopsy samples of additional 27 DLBCL and 3 BL cases. Most of the tested tumor samples showed a high number of CD19- and CD20-positive tumor cells > 70% per case (**Figure 1B**). 3 cases were identified to be CD20-negative, while expression of CD19 on these samples was preserved. Of note, none of these CD20-negative cases had received prior anti-CD20 therapy. In addition, CD19 and CD20 surface expression were determined on a comprehensive panel of 21 DLBCL and 7 BL cell lines to investigate whether these cell lines represent adequate models for functional *in vitro* and *in vivo* assays. These analyses showed that all cell lines expressed both CD19 and CD20 to a various extent in comparison to the negative control T-cell lymphoma cell line FE-PD (**Figure 1C**).

### Direct cytotoxic effects of tafasitamab, rituximab or the combination on aggressive B-cell lymphoma cell lines

To determine direct cytotoxic effects of tafasitamab and rituximab, we performed cell viability assays in 9 cell line models representing different lymphoma subtypes (GCB DLBCL: HT, SU-DHL-4, and SU-DHL-6; ABC DLBCL: OCI-Ly10, TMD8, SU-DHL-2, and U2932; unclassified DLBCL: NU-DUL-1; BL: Ramos). These analyses showed that 1/9 cell lines was primarily sensitive to tafasitamab (NU-DUL-1) while 3/9 models were sensitive to rituximab (U2932, TMD8 and OCI-Ly10) (**Figure 1D**). The combination resulted in decreased cell viability compared to the respective mono treatments in 5/9 cell lines (HT, SU-DHL-4, SU-DHL-6, SU-DHL-2, and Ramos) (**Figure 1D**). Of note, single antibodies significantly decreased viability of SU-DHL-6 cells quickly. Thus, these cells were treated for only 24 hours (instead of 96 hours).

Loewe additivity analysis (32, 33), a reference model to calculate synergy score for combination effect using SynergyFinder (34–36), indicated a synergistic effect between tafasitamab and rituximab at the three highest concentration combinations of 0.5, 5 and 50 nM for cell lines SU-DHL-4 and SU-DHL-6 (synergy score: 25.4-27.2 for SU-DHL-4 or 14.1-16.0 for SU-DHL-6, respectively; **Figure S1A**).

To obtain additional insights into the nature of the cytotoxic effect of both antibodies, we analyzed changes in cell cycle and apoptosis in four cell lines representing different lymphoma subtypes (SU-DHL-4 and SU-DHL-6 [GCB DLBCL]; SU-DHL-2 and U2932 [ABC DLBCL]). Cell cycle analysis revealed a significantly increased accumulation of cells in G0/G1 upon antibody combination treatment for SU-DHL-4 (combination 66.6% vs. tafasitamab 47.0% or rituximab 55.3%, p<0.0001 for both comparisons) and SU-DHL-6 cells (77.6% vs. 62.2% or 65.7%, p=0.003 or p=0.014, respectively), while no effects were observed in U2932 or SU-DHL-2 cells (**Figure S1B**). Apoptosis analysis after 48 hours incubation with the combination resulted in a significantly increased percentage of Annexin-V-positive/PI-negative cells representing early apoptotic cells for SU-DHL-6 cells (47.8% vs. 27.8% or 29.6%, p=0.007 or p=0.012, respectively; **Figure S1C**). For SU-DHL-4 and U2932 cells, rituximab, but not tafasitamab treatment enhanced the rate of apoptosis after 48 hours treatment. In SU-DHL-2 cells, no effects of the antibodies were observed regarding apoptosis (**Figure S1C**).

### NK cell-mediated cytotoxicity of tafasitamab, rituximab or the combination

**Figure 2:**
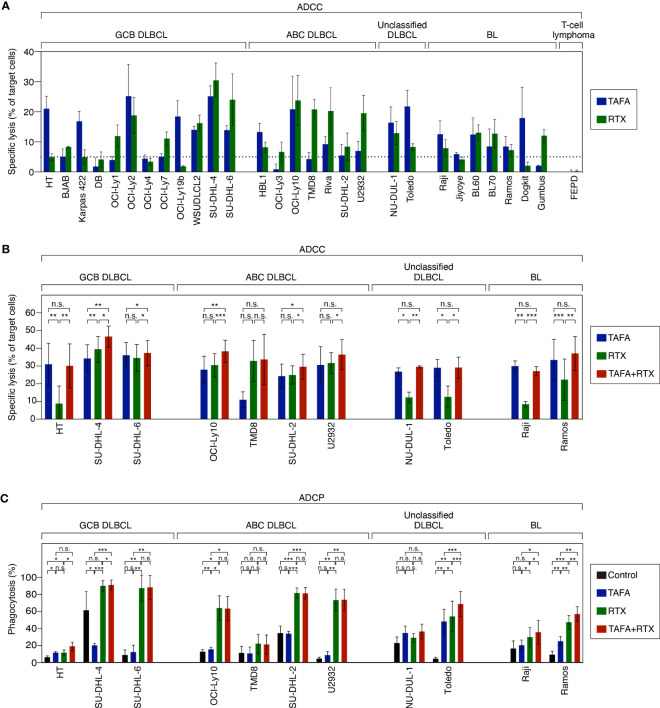
ADCC and ADCP activity of tafasitamab, rituximab and the combination of both antibodies in different B-cell lymphoma cell lines. **(A)** ADCC screen investigating tafasitamab and rituximab: primary NK cells were used as effector cells; 21 DLBCL, 7 BL cell lines and FE-PD (control) were used as target cells; E:T = 1:1. **(B)** ADCC combination experiments: primary NK cells were used as effector cells; 11 cell lines representing the different lymphoma subtypes were used as target cells; The cells were incubated with tafasitamab, rituximab and the combination of both antibodies at concentrations of either 1 or 10 nM of each; E:T ratios = 0.5:1 (HT, SU-DHL-4, and Toledo), 1:1 (OCI-Ly10, TMD8, SU-DHL-2, NU-DUL-1, and Raji), or 3:1 (SU-DHL-6, U2932, and Ramos). Cytotoxicity (%) was determined by quantification of DAPI-positive CFSE-labeled target cells. The graphs show mean values of independent experiments performed with effector cells from 3 to 4 different donors (HT: 4, SU-DHL-4: 4, SU-DHL-6: 3, OCI-LY10: 3, TMD8: 3, SU-DHL-2: 4, U2932: 4, NU-DUL-1: 3, Toledo: 3, Raji: 3, Ramos: 4). **(C)** ADCP assay: DLBCL and BL cells (CellTrace Violet labelled) were used as target cells and monocyte-derived macrophages (CFSE labelled) were used as effector cells; E:T = 2:1. The cells were incubated without antibody (control), with tafasitamab, rituximab or the combination of both antibodies at saturating antibody concentrations of 10 nM; E:T = 2:1. Phagocytosis (%) was determined by quantification of double-positive cells. The graphs show mean values of independent experiments performed with effector cells from 3 to 5 different donors (HT: 3, SU-DHL-4: 4, SU-DHL-6: 3, OCI-LY10: 3, TMD8: 3, SU-DHL-2: 4, U2932: 4, NU-DUL-1: 3, Toledo: 5, Raji: 4, Ramos: 4). Statistical analysis: paired t test. *p<0.05, **p<0.01, ***p<0.001, n.s., not significant.

After determining that both antibodies exert direct cytotoxic effects, we investigated additional modes of action. To compare ADCC effects of tafasitamab vs. rituximab as single agents, a cytotoxicity screen using a panel of 28 lymphoma models (12 GCB, 7 ABC, 2 unclassified DLBCL, and 7 BL cell lines) was performed (**Figure 2A**). We detected that 26/28 cell lines were either primarily sensitive to tafasitamab, rituximab or both antibodies under these experimental conditions (sensitivity threshold: cytotoxicity >5%; **Figure 2A**).

To determine combination ADCC activity, NK cells were co-incubated with either tafasitamab, rituximab or the combination in 11 target cell lines representing the different lymphoma subtypes. In 7/11 cell lines (HT, TMD8, U2932, NU-DUL-1, Toledo, Raji and Ramos), the combination of both antibodies did not outperform the monotherapy activity of each single antibody. More specifically, tafasitamab showed a higher rate of ADCC over rituximab in 5/11 cell lines (HT, NU-DUL-1, Toledo, Raji and Ramos), while tafasitamab and rituximab induced similar cytotoxicity rates in 2/11 cell lines (TMD8 and U2932) (**Figure 2B**). In contrast, in the remaining 4/11 cell lines, significantly increased ADCC activity was observed upon combination treatment compared to the single agents tafasitamab or rituximab (combination 46.6% vs. tafasitamab 34.3% or rituximab 39.5%, p=0.004 or p=0.023 for SU-DHL-4; 46.6% vs. 34.3% or 39.5%, p=0.004 or p=0.023 for SU-DHL-6, 38.3% vs. 28.0% or 30.5%, p=0.007 or p=5×10^-4^ for OCI-Ly10; 30.0% vs. 24.2% or 25.0%, p=0.023 or p=0.03 for SU-DHL-2).

### Macrophage-mediated phagocytosis of tafasitamab, rituximab or the combination

ADCP activity of either antibody alone or the combination was evaluated by co-culturing monocyte-derived macrophages with target cell lines originating from different lymphoma subtypes (**Figure 2C**). The same 11 cell lines, investigated in ADCC assays were tested in ADCP assays. While 4/11 cell lines were sensitive to rituximab (SU-DHL-6, OCI-Ly10, SU-DHL-2 and U2932), 1/11 cell lines showed similar sensitivity to tafasitamab and rituximab (HT). In TMD8 and NU-DUL-1 cells, tafasitamab- and rituximab-mediated effects were not significantly different compared to the control, wherefore these cell lines were categorized as insensitive. In 4/11 cell lines, the combination of tafasitamab and rituximab resulted in significantly enhanced ADCP activity compared with single agent (combination 91.4% vs. tafasitamab 20.0% vs. rituximab 90.2%, p = 0.0002 or p=0.045 for SU-DHL-4, 68.8% vs. 48.2% or 54.2%, p=3×10^-4^ or p=9.0×10^-4^ for Toledo; 35.5% vs. 20.3% or 29.8%, p=0.038 or p=0.034 for Raji; 57.0% vs. 25.0% or 47.3%, p=0.002 of both for Ramos). Of note, an unexpected high phagocytosis rate of 61% was observed in the control samples for SU-DHL-4 cells.

**Figure 3:**
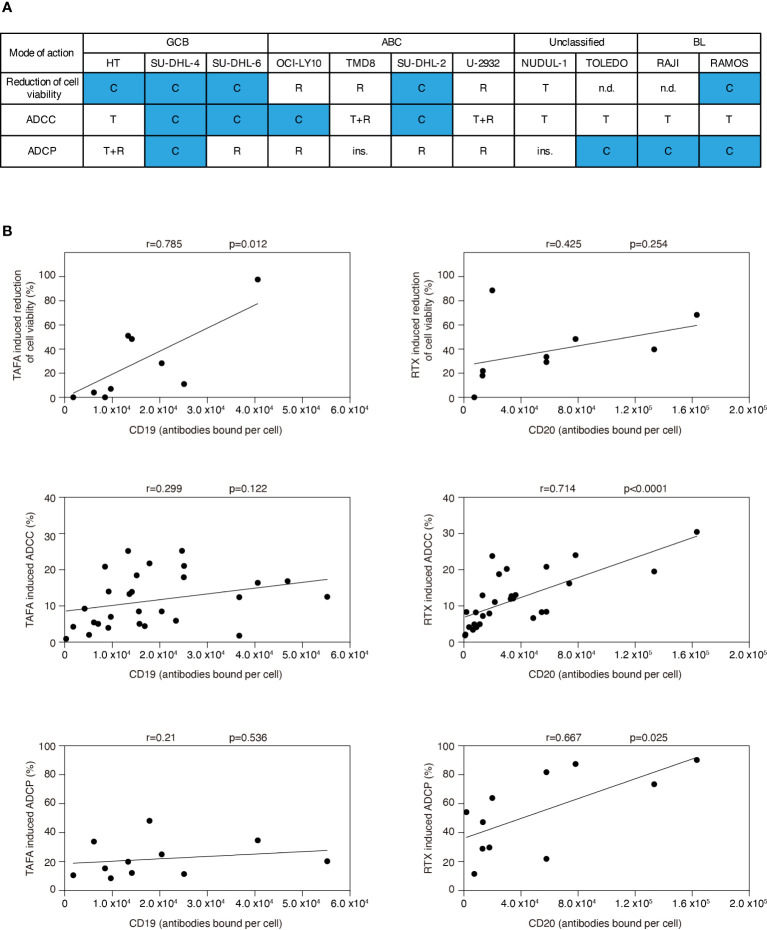
Correlation analyses for efficacy of tafasitamab and rituximab with respect to different modes of action and CD19 or CD20 expression. **(A)** Cell sensitivities to tafasitamab or rituximab alone or combination treatment are indicated. T: Primarily sensitive to tafasitamab; R: Primarily sensitive to rituximab; T+R: Sensitive to both antibodies or no increase in efficacy by combination; C: Increased efficacy through combination of tafasitamab and rituximab; ins.: Insensitive; n.d.: Not determined. **(B)** Pearson’s correlation was performed by determining the relationship between efficacy of tafasitamab or rituximab and CD19 or CD20 expression level, respectively.

In summary, we detected a heterogeneous pattern regarding whether rituximab or tafasitamab or a combination of both antibodies induced higher efficacy of cell viability reduction, ADCC and ADCP (**Figure 3A**). A combination benefit of tafasitamab with rituximab was observed in 8/11 DLBCL or BL cell lines in at least one of the three modes of action tested (HT, SU-DHL-4, SU-DHL-6, OCI-Ly10, SU-DHL-2, Toledo, Raji and Ramos), while one cell line was primarily sensitive to tafasitamab (NU-DUL-1) and two to rituximab (TMD8 and U2932), respectively (**Figure 3A**).

### Correlation between target expression level and antibody activity

To investigate if CD19 or CD20 expression correlated with different modes of action of each antibody, we correlated cell viability, ADCC and ADCP with CD19 and CD20 surface levels (**Figure 3B**). Tafasitamab-mediated reduction of cell viability significantly correlated with CD19 expression (p=0.012) and rituximab-mediated ADCC and ADCP with CD20 expression (p<0.0001 and p=0.025, respectively). No correlation was detectable for tafasitamab-mediated ADCC and ADCP with CD19 expression (p=0.122 and p=0.536, respectively) or rituximab-mediated reduction of cell viability with CD20 expression (p=0.254). Next, we investigated whether molecular lymphoma subtypes correlated with either modes of action of tafasitamab or rituximab. Our analysis indicated no significant correlations (**Supplementary T** **able S2**).

### Gene expression profiling analysis following tafasitamab, rituximab or the combination treatment

**Figure 4:**
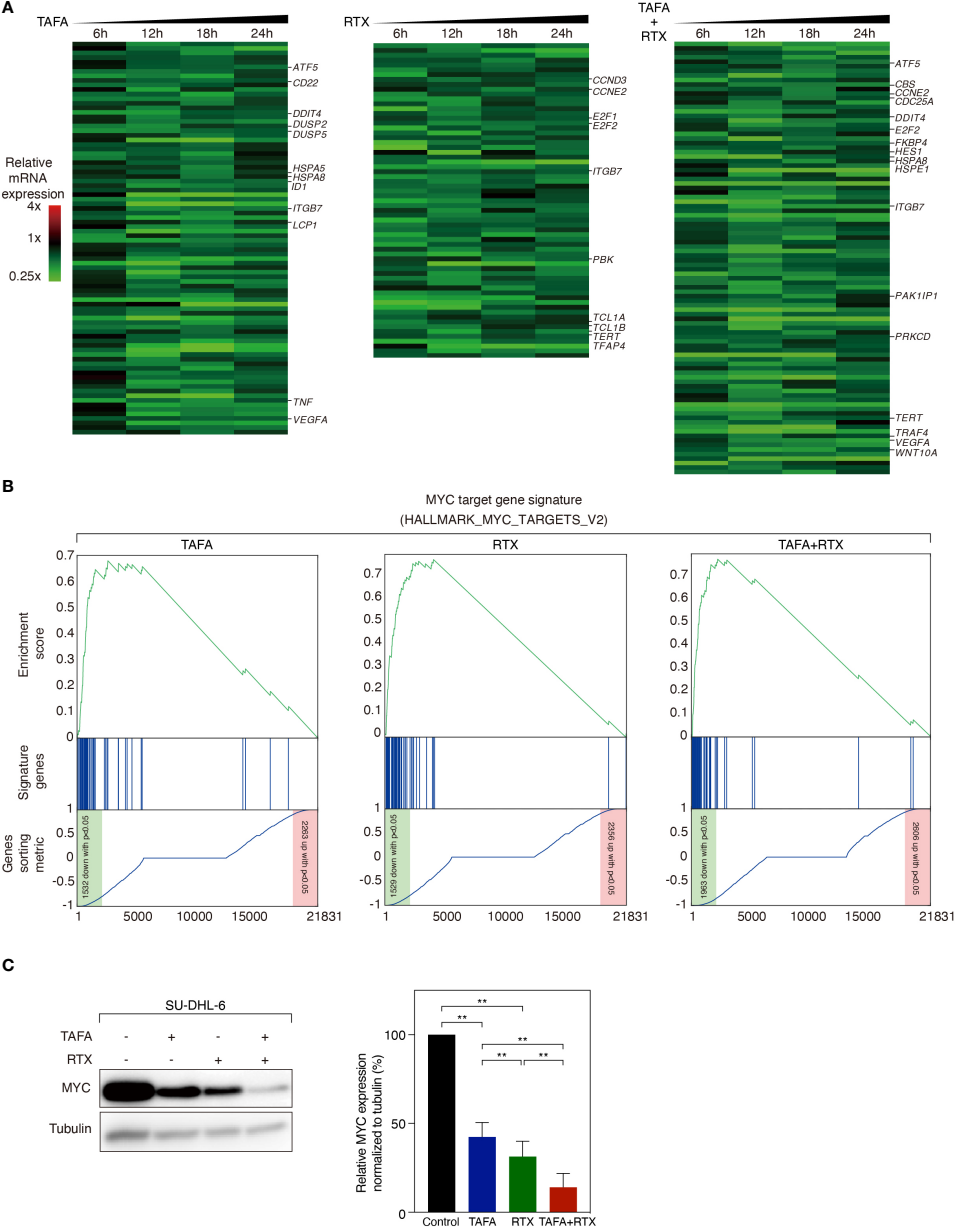
Gene expression profiling analysis following the treatment of tafasitamab or rituximab alone or their combination. **(A)** Gene expression analysis was performed after treatment of SU-DHL-6 cells with 5 nM tafasitamab and/or 5 nM rituximab for 6, 12, 18 and 24 hours. Changes of gene expression are depicted as mean of log2-transformed expression ratios for two replicates according to the color scale shown. Genes that are involved in critical biological processes are highlighted. **(B)** Gene set enrichment analysis of a previously described MYC gene expression signature following the treatment of either tafasitamab or rituximab alone or their combination. **(C)** Protein expression of MYC was assessed after treatment of SU-DHL-6 cells with 5 nM Tafasitamab and/or 5 nM rituximab at 24 hours by western blotting analysis. Representative western blotting results and relative MYC expression normalized to tubulin from three independent replicates are shown. Statistical analysis: paired t test. **p<0.01.

We observed that direct cytotoxic effect of rituximab and tafasitamab was most pronounced in SU-DHL-6 cells. To investigate which signaling cascades are affected by tafasitamab, rituximab and their combination, we performed RNA sequencing following antibody treatment for 6, 12, 18 and 24 hours in SU-DHL-6 cells. We compared gene expression to untreated control samples across all time points. We detected that 86 genes were significantly downregulated after treatment with tafasitamab, 65 genes following treatment with rituximab, and 96 genes following combination treatment (p ≤ 5×10^-7^, non-negative binomial tests; **Figure 4A**). In addition, 97 genes were significantly upregulated after tafasitamab treatment, 107 genes following rituximab treatment, and 132 genes after combination treatment (p ≤ 1×10^-15^, non-negative binomial tests; **Figure S2**). All top gene sets are provided in **Supplementary Tables S3-5**.

**Figure 5:**
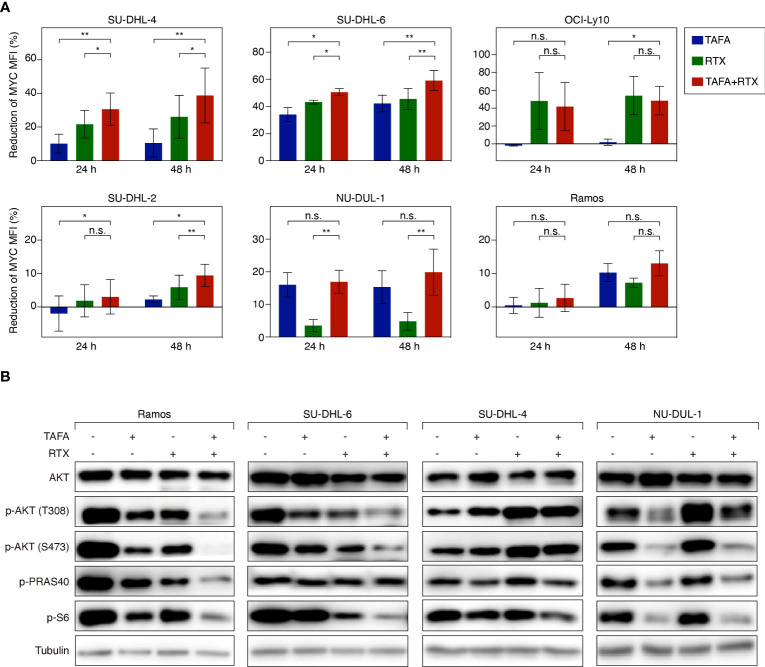
Flow cytometry analysis of MYC expression and western blotting analysis of AKT signaling activity following the treatment of tafasitamab or rituximab alone or their combination treatment. **(A)** MYC expression level of cells was determined after the treatment of 5 nM tafasitamab, 5 nM rituximab or their combination for 24 or 48 hours. MFI of treated cells was analyzed by flow cytometry and normalized to untreated control cells. The graphs depict mean values with SD of two or three independent experiments. Statistical analysis: paired t test. *p<0.05, **p<0.01, n.s. not significant. **(B)** Phosphorylation of AKT, PRAS40 and S6 was measured by western blotting upon treatment of Ramos, SU-DHL-6, SU-DHL-4, and NU-DUL-1 cells with 0.05 nM (SU-DHL-6) or 5 nM (other cell lines) tafasitamab, 5 nM rituximab or their combination for 24 hours. Representative western blotting results from at least 3 independent replicates are shown.

To systematically analyze in an unbiased fashion which molecular pathways are affected by tafasitamab, rituximab or the combination of both antibodies, we performed a gene set enrichment analysis (GSEA) using a database comprised of 23.026 published signatures (23, 28–31). We observed that various previously described MYC target gene signatures were significantly downregulated following tafasitamab or rituximab treatment alone or their combination in SU-DHL-6 cells, suggesting inhibition of the MYC regulated gene expression network (**Supplementary Table S6**; **Figure 4B**). Interestingly, MYC mRNA was not significantly downregulated following treatment with tafasitamab, rituximab or the antibody combination (tafasitamab: Log2 ratio= 0.0545; rituximab: Log2 ratio= -0.3213; combination: Log2 ratio= -0.2313). Thus, we reasoned that MYC might be predominantly regulated at the posttranscriptional level. To verify our hypothesis, protein expression of MYC was determined by western blotting following tafasitamab and/or rituximab in SU-DHL-6 cells after 24 hours. Tafasitamab or rituximab alone resulted in downregulation of MYC protein expression which was further enhanced when the two antibodies were combined (**Figure 4C**). These results were confirmed for SU-DHL-6 cells in an intracellular flow cytometry assay detecting decreased MYC expression following antibody treatment for 24 or 48 hours (**Figure 5A**). Using the same assay, largest MYC downregulation following combination treatment was detected for SU-DHL-4, SU-DHL-2 and SU-DHL-6 cells. In OCI-Ly10 and NU-DUL-1 cells, decrease of MYC levels appeared to be induced by rituximab and tafasitamab monotherapies, respectively (**Figure 5A**). In summary, these data suggest that the direct cytotoxic effects of tafasitamab or rituximab treatment are at least partially caused by downregulation of MYC at the posttranscriptional level resulting in downregulation of its target genes.

### AKT pathway activity following tafasitamab, rituximab or the combination treatment

Previous work has shown that MYC expression can be regulated among others by B-cell receptor (BCR) signaling (37). It has also been reported that rituximab can inhibit BCR signaling and downstream signaling cascades such as the AKT pathway (38). To this end, we aimed to investigate if tafasitamab or the combination of tafasitamab and rituximab inhibits BCR signaling and its downstream targets such as AKT, PRAS40 and S6, which in turn would result in downregulation of MYC expression. Tafasitamab as well as rituximab treatment of Ramos and SUDHL-6 cells resulted in decreased phosphorylated level of AKT (T308 and S473) as well as phosphorylated level of S6 compared to untreated samples (**Figure 5B**). When the two antibodies were combined, further reduction of target phosphorylation was detectable suggesting a stronger inhibition of constitutive AKT/mTOR signaling in both cell lines. Interestingly, analysis of SU-DHL-4 cells revealed an opposite effect for rituximab compared to SU-DHL-6 and Ramos cells, as phosphorylation of AKT (T308 and S473) was increased and not decreased upon rituximab treatment, while a minor increase was observed for p-AKT upon tafasitamab monotherapy, and no additional increase found for the antibody combination. Consistently, NU-DUL-1 cells, which were identified as sensitive to tafasitamab but not to rituximab in our cell viability assays, demonstrated decreased phosphorylation of AKT pathway components compared to untreated cells following treatment with tafasitamab but not after rituximab (**Figure 5B**).

### *In vivo* activity of tafasitamab, rituximab and the combination

**Figure 6:**
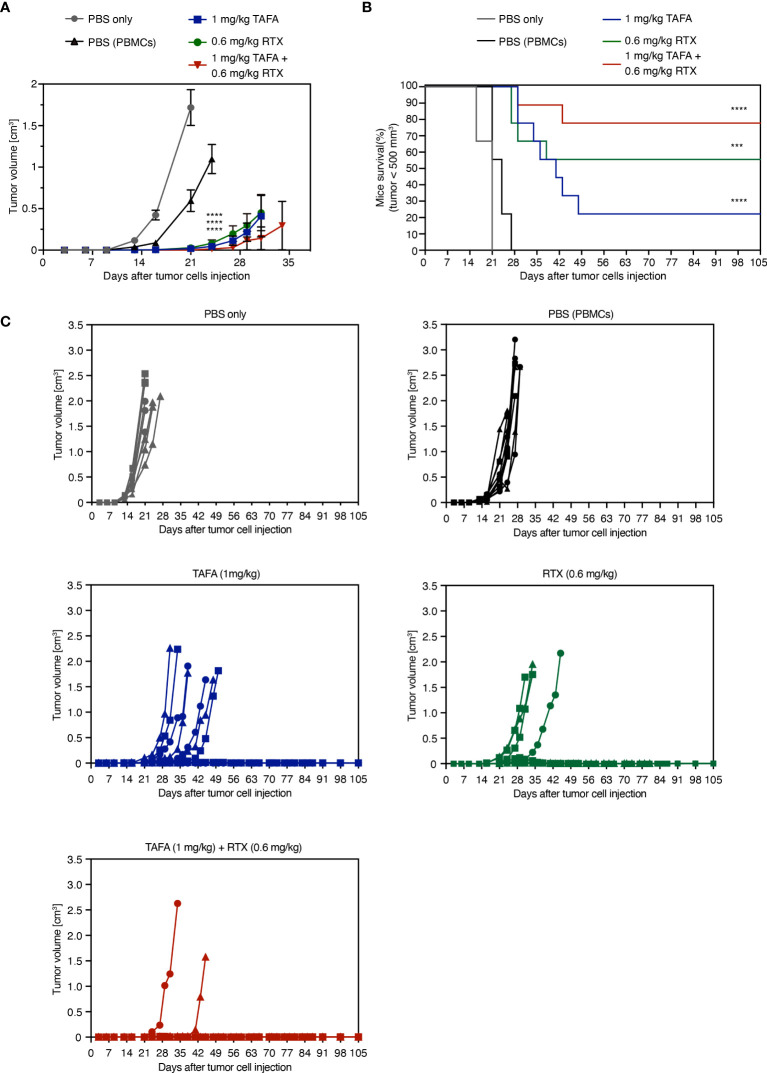
*In vivo* activity of tafasitamab, rituximab and the combination in a PBMC-humanized Ramos mouse model. **(A, B)** Ramos cells and PBMC were co-engrafted subcutaneously with an E:T ratio of 5:1 in NOD/SCID mice on the same day, when intraperitoneal treatment was initiated. PBMC from three different donors were engrafted in 9 mice per group (3 mice per donor and group). Mice were treated twice weekly for six weeks in total. Graphs depict mean tumor volumes **(A)** and survival rate **(B)** of mice treated with 1 mg/kg tafasitamab, 0.6 mg/kg rituximab or both of antibodies. For the two control groups, mice were either engrafted with Ramos cells only and treated with PBS (PBS only) or co-engrafted with Ramos cells plus PBMC and treated with PBS (PBS(PBMCs)). Kaplan-Meier curve depicts the time point at which tumor volume has reached 500 mm^3^. On day 105, 7/9 mice were alive in the combination-treated group, while 2/9 mice were alive in the tafasitamab-treated and 5/9 in the rituximab-treated group. **(C)** Individual tumor volumes per group determined until day 105 are shown. Different symbols represent different PBMC donors. Mann-Whitney or Mantel-Cox tests were performed to determine tumor volume differences or mice survival trends between groups, respectively. ***p<0.001, ****p<0.0001, n.s. not significant, vs. PBMC control.

Next, we determined if our *in vitro* results translate into an *in vivo* setting. Tafasitamab or rituximab alone reduced the size of Ramos tumors in comparison to vehicle treatment with PBMC to various extents, depending on the applied antibody concentrations (**Figures 6A, C**, **S3A, S3C**). When 1 mg/kg tafasitamab and 0.6 mg/kg rituximab were applied, the combination treatment led to further statistically significant decrease of mean tumor volume compared to the mono treatment of tafasitamab, while a similar trend was observed compared to rituximab (p=0.032 and p=0.079, respectively; **Figure 6A, C**). Other combination regimens of different doses of both antibodies resulted in efficacies outperforming one or the other mono arms, but not both (**Figures S3A, C**).

Tumor growth was monitored until day 105 post implantation: 7/9 animals remained tumor free until this timepoint in the combination group, compared to 5/9 in the rituximab and 2/9 in the tafasitamab monotherapy groups. Combined application of tafasitamab and rituximab also resulted in a statistically significant survival benefit compared to tafasitamab alone (combination vs. tafasitamab or rituximab, p=0.022 or 0.27, respectively; **Figure 6B**). Alternative combination regimens yielded significance over both (**Figure S3B**) or the rituximab mono arm only (**Figure S3D**). At last, the survival data were analyzed using a mixed effect multivariable Cox proportional hazards model to account for potential synergy or additivity between tafasitamab and rituximab (**Supplementary Table** **S7**) (39–41). The method confirmed synergy between 1 mg/kg tafasitamab and 0.6 mg/kg rituximab. Additivity was confirmed for a combination of 1 mg/kg tafasitamab and 0.3 mg/kg rituximab.

## Discussion

In this study we explored the scientific rationale of the combination tafasitamab and rituximab in aggressive B cell lymphoma. To our knowledge, this is the first study to comprehensively dissect the combination potential of these two antibodies, currently being used for the treatment of relapsed/refractory DLBCL (10, 42–44).

We investigated three reported mechanisms of action of tafasitamab and rituximab: direct cytotoxicity, ADCC and ADCP. Compared to the activities of the single antibodies, their combination exhibited further additive or synergistic effects which were cell line-dependent in either mode of action. Reduced cell viability was detectable as a combination effect of tafasitamab and rituximab in 5/9 cell lines, whereas one cell line weas primarily sensitive to tafasitamab and three to rituximab. Both antibodies were demonstrated to cooperate in inducing ADCC in 4/11 and ADCP in 4/11 cell lines. Altogether, the combination of tafasitamab and rituximab might be beneficial in three different modes of action: direct cytotoxicity, ADCC and ADCP.

Furthermore, correlation analyses of tafasitamab- and rituximab-mediated effects by mode of action with CD19 or CD20 expression levels were conducted. Interestingly, in 3/6 analyses, significant correlations were observed, while in the remaining three no significant correlations were detected: as described, tafasitamab-mediated reduction of cell viability significantly correlated with CD19 expression and rituximab-mediated ADCC and ADCP with CD20 expression. Based on these observations it can be hypothesized that antigen expression level is likely not the only factor determining antibody-mediated anti-tumor activity. There might be additional factors, negatively or positively influencing antibody-induced anti-tumor effects. For instance, ADCC and ADCP can be modulated by differential expression of regulatory molecules by the target and the effector cells (45–48). Further studies are required to address the relative role of antigen expression in the context of antibody-mediated effects.

On a molecular level, the effects of tafasitamab and rituximab on MYC provide a potential explanation for the observed cooperation between both antibodies in reducing cell viability. The extensively studied oncogene MYC, able to control essential cellular processes such as proliferation, is dysregulated in a variety of B-cell lymphomas (24, 49–52). Previous studies on the BCR signaling pathway have linked MYC to the mode of action of rituximab. MYC activation can be decreased via BCR signaling downregulation, while rituximab can inhibit BCR downstream cascades via downmodulation of the AKT pathway (37, 38). Our results in SU-DHL-6 cells suggest that tafasitamab may, like rituximab, inhibit AKT signaling and thus induce direct cytotoxicity in a similar manner, with net effects of the combination translating into a stronger cytotoxic potential. However, we could not confirm these results in SU-DHL-4 cells which showed enhanced AKT signaling upon antibody treatment, consistent with previously reported data following treatment with rituximab (53). Yet, the sensitivity of SU-DHL-4 to direct killing may be explained with the existence of certain thresholds for BCR signaling (54, 55). When critical survival signals fall below a minimum threshold, or when signaling is hyperactivated above a maximum threshold, cell death is induced consequently. According to this concept, SU-DHL-6 and Ramos cells treated with tafasitamab and/or rituximab would die due to AKT signaling inhibition below a certain threshold, while SU-DHL-4 cells would die due to hyperactivated AKT signaling. Collectively, our findings suggest that the biological processes underlying the mechanisms of action and cooperation of tafasitamab and rituximab are very complex and may differ in different cellular systems. Thus, further work is needed to fully unravel the molecular mechanisms behind the growth inhibitory effect of the combination. Finally, we were able to confirm our *in vitro* findings in a PBMC-humanized lymphoma *in vivo* model. These results are in line with the study by Ward et al., which demonstrated that combination treatment with an anti-CD19 antibody and rituximab resulted in decreased tumor growth in lymphoma mouse models compared to the respective mono-treatments (56). Importantly, certain dosing combinations achieved synergistic anti-tumor efficacy *in vivo*, thus highlighting the benefit of a joint treatment in a system where all different modes of action of both antibodies can be at play.In summary, our study indicates that the combination of tafasitamab and rituximab has the potential to eliminate tumor cells cooperatively and complementarily across different lymphoma subtypes *in vitro* and *in vivo*. These results are of high translational interest, as they provide a biological rationale to combine tafasitamab and rituximab in a clinical trial setting.

## Data availability statement

The datasets presented in this study can be found in online repositories. The names of the repository/repositories and accession number(s) can be found below: NCBI via accession ID: GSE212829.

## Ethics statement

All human material was obtained with written informed consent and its use was approved by the local Institutional Ethical Review Boards (2018-15 452-f-S).

## Author contributions

MP-K, GC, CH, JE and GL conceived of the presented idea. WX, CA, MG, MZ, KI, KL, DM-E, KY, PB, KK, GO, PK, CK, PH, JS and SS contributed to the experimental design, the data acquisition, analysis, interpretation, and conceptualization. MP wrote the manuscript. KI, CH, SS, GC and GL contributed to editing and finalization of the text. GL supervised the project. All authors contributed to the article and approved the submitted version.
