# Supplementary material for: Activity of tafasitamab in combination with rituximab in subtypes of aggressive lymphoma
Source: Front Immunol. 2023 Jul 31;14:1220558. doi: 10.3389/fimmu.2023.1220558 (PMC10433160; doi:10.3389/fimmu.2023.1220558)
Supplement: Supplementary file 1 [file DataSheet_1.docx]

Supplementary Material

Activity of tafasitamab in combination with rituximab in subtypes of aggressive lymphoma

Maria Patra-Kneuer^1†^, Gaomei Chang^2†^, Wendan Xu^2^, Christian Augsberger^1^, Michael Grau^2^, Myroslav Zapukhlyak^2^, Kristina Ilieva^1^, Karin Landgraf^1^, Doris Mangelberger-Eberl^1^, Kasra Yousefi^1^, Philipp Berning^2^, Katrin S. Kurz^3^, German Ott^3^, Pavel Klener^4,5^, Cyrus Khandanpour^2,6^, Pedro Horna^7^, Jürgen Schanzer^1^, Stefan Steidl^1^, Jan Endell^1^, Christina Heitmüller^1^, Georg Lenz^2*^

^1^ MorphoSys AG, Planegg, Germany

^2^ Department of Medicine A, Hematology, Oncology and Pneumology, University Hospital Münster, Münster, Germany

^3^Department of Clinical Pathology, Robert-Bosch-Krankenhaus and Dr. Margarete Fischer-Bosch Institute for Clinical Pharmacology, Stuttgart, Germany

^4^Institute of Pathological Physiology, First Faculty of Medicine, Charles University Prague, Prague, Czech Republic

^5^First Medical Department, Department of Hematology, Charles University General Hospital Prague, Prague, Czech Republic.

^6^ Hematology and Oncology Clinic, University of Lübeck and University Hospital Schleswig-Holstein, Lübeck, Germany

^7^ Division of Hematopathology, Mayo Clinic, Rochester, MN, USA

† These authors contributed equally to this work

*** Correspondence:**Prof. Georg Lenz
georg.lenz@ukmuenster.de

# Supplementary Methods

**Cell cycle assay and Annexin-V staining**

B-cell lymphoma cell lines were incubated with 10 nM tafasitamab, 10 nM rituximab or the combination of both for 36 hours. Cell cycle assay was performed using the NucleoCounter NC250 (Chemometec) according to a two-step cell cycle analysis. The distributions of the cell cycle were analyzed by ModFit LT. Annexin-V staining was performed after treatment of the cells with 10 nM tafasitamab, 10 nM rituximab or the combination of both antibodies for 24 or 48 hours using the FITC Annexin-V Apoptosis Detection Kit I (Beckton Dickinson) according to the manufacturers’ protocol.

**Synergy analysis for in vitro and in vivo studies**

Synergy analysis of cell viability data was performed by applying the Loewe additivity model to the drug combination treatments data for selected concentration tuples using SynergyFinder v3.2.2 package in R v4.1.1 with RStudio v1.4.1717 (1-3).

The statistical analyses of mice survival to evaluate additive or synergistic effects upon combination of tafasitamab and rituximab were performed in R v4.0. All statistical tests were two-sided using a 5% significance level. For the survival analysis endpoint, a mixed effect multivariable Cox proportional hazards model was fitted (4-6). Partial likelihood ratio test was performed to assess the significance of each of the main effects and the relevant interaction term between tafasitamab and rituximab. Main effects included PBS (PBMCs), tafasitamab and rituximab treatment. The interaction term was fitted for tafasitamab and rituximab. PBMCs donor was fitted as a random effect to account for donor variations. Three separate models were fitted for each combination. Per tafasitamab, rituximab and combination were found to be additive if the main effect for each term was significant, but the interaction term was not significant. Tafasitamab and rituximab were considered synergistic if the main effect for each term was significant and the interaction term was found to be significant (4-6).

**Statistical analysis**

Multiple comparisons analysis for cell cycle and apoptosis assay was performed with one-way ANOVA. Statistical analyses were performed with GraphPad Prism (RRID:SCR_002798) versions 8 or 9 software. Multiple comparison analysis for the cytotoxicity in cell lines grouped by lymphoma type was performed using the Kruskal-Wallis test (Statistics and Machine Learning Toolbox of MATLAB® R2021a, The MathWorks® Inc.).

**References**

1. Zheng S, Wang W, Aldahdooh J, Malyutina A, Shadbahr T, Tanoli Z, et al. Synergyfinder Plus: Toward Better Interpretation and Annotation of Drug Combination Screening Datasets. *Genom Proteom Bioinform* (2022) 20(3):587-96. Epub 2022/01/28. doi: 10.1016/j.gpb.2022.01.004.

2. He L, Kulesskiy E, Saarela J, Turunen L, Wennerberg K, Aittokallio T, et al. Methods for High-Throughput Drug Combination Screening and Synergy Scoring. *Methods Mol Biol* (2018) 1711:351-98. Epub 2018/01/19. doi: 10.1007/978-1-4939-7493-1_17.

3. Ianevski A, He L, Aittokallio T, Tang J. Synergyfinder: A Web Application for Analyzing Drug Combination Dose-Response Matrix Data. *Bioinformatics* (2017) 33(15):2413-5. Epub 2017/04/06. doi: 10.1093/bioinformatics/btx162.

4. Christensen E. Multivariate Survival Analysis Using Cox's Regression Model. *Hepatology* (1987) 7(6):1346-58. doi: 10.1002/hep.1840070628.

5. Terry M. Therneau PMG. *Modeling Survival Data: Extending the Cox Model*(2000).

6. D.R. Cox DO. Analysis of Survival Data. (1984). doi: <https://doi.org/10.1201/9781315137438>.

**Supplementary Figures**

**Supplementary Figure S1.**

**Synergy analysis for cell viability. (A)** SU-DHL-4 and SU-DHL-6 cells were treated with different concentrations of tafasitamab and/or rituximab for 96 hours or 24 hours, respectively. Cell viability was determined using the Cell-Titer Glo assay and normalized to untreated cells. The graphs (left) show mean values with SEM of 3 independent experiments. Synergy analysis (right) was performed with Loewe method and depicted as synergy score according to the color scale shown. **(B)** Cell cycle distribution after treatment with 10 nM tafasitamab and/or 10 nM rituximab was measured at 36 hours. Data are shown as mean values with SD of three independent experiments. **(C)** Cells were treated with 10 nM tafasitamab and/or 10 nM rituximab for 24 hours and 48 hours. Early apoptosis is shown as percentage of Annexin-V-positive/PI-negative cells using flow cytometry. Data are shown as mean values with SD of three independent experiments. Statistical analysis: One-way ANOVA. *p<0.05, **p<0.01, ***p<0.001, ****p<0.0001, n.s. not significant.

**Supplementary Figure S2.**

**Gene expression profiling analysis.** Gene expression profiling analysis was performed after treatment of SU-DHL-6 cells with 5 nM tafasitamab and/or 5 nM rituximab for 6, 12, 18 and 24 hours. Changes of gene expression are depicted according to the color scale shown. Left to right, 97, 107 and 132 genes respectively were identified as significantly (p ≤ 1x10^-15^) upregulated across all time points after treatment of tafasitamab, rituximab or combination of both antibodies, respectively.

**Supplementary Figure S3.**

**In vivo activity of tafasitamab, rituximab and the combination of both antibodies in a PBMC-humanized Ramos mouse model. (A** and **C)**, Graphs depict tumor volumes of mice treated with either 0.3 **(A)** or 1 mg/kg **(C)** tafasitamab, 0.3 mg/kg rituximab or both of antibodies, respectively. Mann-Whitney test was performed to determine mice tumor volume differences between groups (day 21 for A, day 24 for C). *p<0.05, **p<0.01, ***p<0.001, ****p<0.0001, n.s. not significant, vs. PBMCs control. Combination (**A**) vs. tafasitamab or rituximab, p=0.005 or p=0.053, respectively. Combination (**C**) vs. tafasitamab or rituximab, p=0.09 or p=0.019, respectively. (**B** and **D**) Survival analysis of mice treated with either 0.3 (**B**) or 1 mg/kg (**D**) tafasitamab, 0.3 mg/kg rituximab or both antibodies. Mantel-Cox test was performed to analyze mice survival trends between groups. *p<0.05, **p<0.01, ***p<0.001, ****p<0.0001, n.s. not significant, vs. PBMCs control. Combination (**B**) vs. tafasitamab or rituximab, p=0.015 or p=0.033, respectively. Combination (**D**) vs. tafasitamab or rituximab, p=0.333 or p=0.007, respectively.

**Supplementary Tables**

**Supplementary Table S1**

| **Lymphoma cell lines** | | | | |
| --- | --- | --- | --- | --- |
| Cell line | Disease | Identity | Identified by GEP or previous publications | Culture medium |
| HT | DLBCL | GCB | Tabea Erdmann, *et al*., Blood 2017 | RPMI 1640 + 10%FCS |
| BJAB | DLBCL | GCB | Tabea Erdmann, *et al*., Blood 2017 | RPMI 1640 + 10%FCS |
| Karpas422 | DLBCL | GCB | Tabea Erdmann, *et al*., Blood 2017 | RPMI 1640 + 10%FCS |
| DB | DLBCL | GCB | Tabea Erdmann, *et al*., Blood 2017 | RPMI 1640 + 10%FCS |
| OCI-Ly1 | DLBCL | GCB | Tabea Erdmann, *et al*., Blood 2017 | IMDM + 10%FCS |
| OCI-Ly2 | DLBCL | GCB | Tabea Erdmann, *et al*., Blood 2017 | IMDM + 10%FCS |
| OCI-Ly4 | DLBCL | GCB | Tabea Erdmann, *et al*., Blood 2017 | IMDM + 10%FCS |
| OCI-Ly7 | DLBCL | GCB | Hendrik Nogai, *et al.*, Bood 2013 | IMDM + 10%FCS |
| OCI-Ly19 | DLBCL | GCB | Tabea Erdmann, *et al*., Blood 2017 | IMDM + 20% human plasma |
| WSUDLCL-2 | DLBCL | GCB | Tabea Erdmann, *et al*., Blood 2017 | RPMI 1640 + 10%FCS |
| SU-DHL-4 | DLBCL | GCB | Tabea Erdmann, *et al*., Blood 2017 | RPMI 1640 + 10%FCS |
| SU-DHL-6 | DLBCL | GCB | Hendrik Nogai, *et al*., Bood 2013 | RPMI 1640 + 20%FCS |
| HBL1 | DLBCL | ABC | Tabea Erdmann, *et al*., Blood 2017 | RPMI 1640 + 20%FCS |
| OCI-Ly3 | DLBCL | ABC | Tabea Erdmann, *et al*., Blood 2017 | IMDM + 20% human plasma |
| OCI-Ly10 | DLBCL | ABC | Tabea Erdmann, *et al*., Blood 2017 | IMDM + 20% human plasma |
| TMD8 | DLBCL | ABC | Tabea Erdmann, *et al*., Blood 2017 | IMDM + 20% human plasma |
| Riva | DLBCL | ABC | Nanostring | RPMI 1640 + 10%FCS |
| SU-DHL2 | DLBCL | ABC | Nanostring | RPMI 1640 + 10%FCS |
| U2932 | DLBCL | ABC | Tabea Erdmann, et al., Blood 2017 | RPMI 1640 + 10%FCS |
| NU-DUL-1 | DLBCL | Unclassified | Nanostring | RPMI 1640 + 20%FCS |
| Toledo | DLBCL | Unclassified | Nanostring | RPMI 1640 + 20%FCS |
| Raji | BL | - | - | RPMI 1640 + 10%FCS |
| Jiyoye | BL | - | - | RPMI 1640 + 10%FCS |
| BL60 | BL | - | - | RPMI 1640 + 20%FCS |
| BL70 | BL | - | - | RPMI 1640 + 20%FCS |
| Ramos | BL | - | - | RPMI 1640 + 10%FCS |
| Dogkit | BL | - | - | RPMI 1640 + 10%FCS |
| Gumbus | BL | - | - | RPMI 1640 + 10%FCS |
| FE-PD | T-cell lymphoma | - | - | RPMI 1640 + 20%FCS |

**Supplementary Table S2**

| **Correlation analysis for lymphoma subtypes and per mode of action of tafasitamab or rituximab** | | | | | | |
| --- | --- | --- | --- | --- | --- | --- |
| Assay | Treatment | Type of test | Significance level alpha | Hypothesis test result h | P value of the test result | Kruskal-Wallis/chi-square statistics value |
| Reduction of cell viability | Tafasitamab | Kruskal-Wallis | 0,05 | 0 | 0,082 | 6,711 |
| ADCC | Tafasitamab | Kruskal-Wallis | 0,05 | 0 | 0,321 | 3,498 |
| ADCP | Tafasitamab | Kruskal-Wallis | 0,05 | 0 | 0,113 | 5,962 |
| Reduction of cell viability | Rituximab | Kruskal-Wallis | 0,05 | 0 | 0,510 | 2,311 |
| ADCC | Rituximab | Kruskal-Wallis | 0,05 | 0 | 0,387 | 3,029 |
| ADCP | Rituximab | Kruskal-Wallis | 0,05 | 0 | 0,707 | 1,394 |

**Supplementary Table S3**

| **Top genes after the treatment of 5 nM tafasitamab in SU-DHL-6** | | | |
| --- | --- | --- | --- |
| Signature name | Gene symbol | GeneID | Gene description |
| Downregulated genes (alpha=5e-7) | *ABI3* | 51225 | ABI family member 3 |
| Downregulated genes (alpha=5e-7) | *ALPL* | 249 | alkaline phosphatase, biomineralization associated |
| Downregulated genes (alpha=5e-7) | *AMIGO2* | 347902 | adhesion molecule with Ig like domain 2 |
| Downregulated genes (alpha=5e-7) | *ANKRD33B* | 651746 | ankyrin repeat domain 33B |
| Downregulated genes (alpha=5e-7) | *ANXA6* | 309 | annexin A6 |
| Downregulated genes (alpha=5e-7) | *ATF5* | 22809 | activating transcription factor 5 |
| Downregulated genes (alpha=5e-7) | *BHLHE40* | 8553 | basic helix-loop-helix family member e40 |
| Downregulated genes (alpha=5e-7) | *CD180* | 4064 | CD180 molecule |
| Downregulated genes (alpha=5e-7) | *CD22* | 933 | CD22 molecule |
| Downregulated genes (alpha=5e-7) | *CD72* | 971 | CD72 molecule |
| Downregulated genes (alpha=5e-7) | *CHCHD10* | 400916 | coiled-coil-helix-coiled-coil-helix domain containing 10 |
| Downregulated genes (alpha=5e-7) | *CHURC1-FNTB* | 100529261 | CHURC1-FNTB readthrough |
| Downregulated genes (alpha=5e-7) | *CLEC17A* | 388512 | C-type lectin domain containing 17A |
| Downregulated genes (alpha=5e-7) | *CREM* | 1390 | cAMP responsive element modulator |
| Downregulated genes (alpha=5e-7) | *CTSZ* | 1522 | cathepsin Z |
| Downregulated genes (alpha=5e-7) | *DDIT4* | 54541 | DNA damage inducible transcript 4 |
| Downregulated genes (alpha=5e-7) | *DDN* | 23109 | dendrin |
| Downregulated genes (alpha=5e-7) | *DENND3* | 22898 | DENN domain containing 3 |
| Downregulated genes (alpha=5e-7) | *DUSP2* | 1844 | dual specificity phosphatase 2 |
| Downregulated genes (alpha=5e-7) | *DUSP5* | 1847 | dual specificity phosphatase 5 |
| Downregulated genes (alpha=5e-7) | *FAM9C* | 171484 | family with sequence similarity 9 member C |
| Downregulated genes (alpha=5e-7) | *FCER2* | 2208 | Fc fragment of IgE receptor II |
| Downregulated genes (alpha=5e-7) | *GCNT1* | 2650 | glucosaminyl (N-acetyl) transferase 1 |
| Downregulated genes (alpha=5e-7) | *GM2A* | 2760 | GM2 ganglioside activator |
| Downregulated genes (alpha=5e-7) | *GPR18* | 2841 | G protein-coupled receptor 18 |
| Downregulated genes (alpha=5e-7) | *HIVEP3* | 59269 | HIVEP zinc finger 3 |
| Downregulated genes (alpha=5e-7) | *HLA-DQA1* | 3117 | major histocompatibility complex, class II, DQ alpha 1 |
| Downregulated genes (alpha=5e-7) | *HLA-DQA2* | 3118 | major histocompatibility complex, class II, DQ alpha 2 |
| Downregulated genes (alpha=5e-7) | *HSPA5* | 3309 | heat shock protein family A (Hsp70) member 5 |
| Downregulated genes (alpha=5e-7) | *HSPA8* | 3312 | heat shock protein family A (Hsp70) member 8 |
| Downregulated genes (alpha=5e-7) | *ID1* | 3397 | inhibitor of DNA binding 1, HLH protein |
| Downregulated genes (alpha=5e-7) | *IFI30* | 10437 | IFI30 lysosomal thiol reductase |
| Downregulated genes (alpha=5e-7) | *IL17RB* | 55540 | interleukin 17 receptor B |
| Downregulated genes (alpha=5e-7) | *IL4I1* | 259307 | interleukin 4 induced 1 |
| Downregulated genes (alpha=5e-7) | *IQSEC1* | 9922 | IQ motif and Sec7 domain ArfGEF 1 |
| Downregulated genes (alpha=5e-7) | *ITGB2* | 3689 | integrin subunit beta 2 |
| Downregulated genes (alpha=5e-7) | *ITGB7* | 3695 | integrin subunit beta 7 |
| Downregulated genes (alpha=5e-7) | *KMO* | 8564 | kynurenine 3-monooxygenase |
| Downregulated genes (alpha=5e-7) | *LAT2* | 7462 | linker for activation of T cells family member 2 |
| Downregulated genes (alpha=5e-7) | *LCP1* | 3936 | lymphocyte cytosolic protein 1 |
| Downregulated genes (alpha=5e-7) | *LMNA* | 4000 | lamin A/C |
| Downregulated genes (alpha=5e-7) | *LMO2* | 4005 | LIM domain only 2 |
| Downregulated genes (alpha=5e-7) | *LRRC32* | 2615 | leucine rich repeat containing 32 |
| Downregulated genes (alpha=5e-7) | *LTA* | 4049 | lymphotoxin alpha |
| Downregulated genes (alpha=5e-7) | *LY86* | 9450 | lymphocyte antigen 86 |
| Downregulated genes (alpha=5e-7) | *LYAR* | 55646 | Ly1 antibody reactive |
| Downregulated genes (alpha=5e-7) | *MANF* | 7873 | mesencephalic astrocyte derived neurotrophic factor |
| Downregulated genes (alpha=5e-7) | *MGLL* | 11343 | monoglyceride lipase |
| Downregulated genes (alpha=5e-7) | *MPEG1* | 219972 | macrophage expressed 1 |
| Downregulated genes (alpha=5e-7) | *NAB2* | 4665 | NGFI-A binding protein 2 |
| Downregulated genes (alpha=5e-7) | *NAPSA* | 9476 | napsin A aspartic peptidase |
| Downregulated genes (alpha=5e-7) | *NAPSB* | 256236 | napsin B aspartic peptidase, pseudogene |
| Downregulated genes (alpha=5e-7) | *NCR2* | 9436 | natural cytotoxicity triggering receptor 2 |
| Downregulated genes (alpha=5e-7) | *NXPH4* | 11247 | neurexophilin 4 |
| Downregulated genes (alpha=5e-7) | *PCDHGC3* | 5098 | protocadherin gamma subfamily C, 3 |
| Downregulated genes (alpha=5e-7) | *PDE9A* | 5152 | phosphodiesterase 9A |
| Downregulated genes (alpha=5e-7) | *PHACTR1* | 221692 | phosphatase and actin regulator 1 |
| Downregulated genes (alpha=5e-7) | *PLD4* | 122618 | phospholipase D family member 4 |
| Downregulated genes (alpha=5e-7) | *PLEK* | 5341 | pleckstrin |
| Downregulated genes (alpha=5e-7) | *PPP1R15A* | 23645 | protein phosphatase 1 regulatory subunit 15A |
| Downregulated genes (alpha=5e-7) | *PTPN6* | 5777 | protein tyrosine phosphatase non-receptor type 6 |
| Downregulated genes (alpha=5e-7) | *QPCT* | 25797 | glutaminyl-peptide cyclotransferase |
| Downregulated genes (alpha=5e-7) | *RGS16* | 6004 | regulator of G protein signaling 16 |
| Downregulated genes (alpha=5e-7) | *RGS2* | 5997 | regulator of G protein signaling 2 |
| Downregulated genes (alpha=5e-7) | *SCARB1* | 949 | scavenger receptor class B member 1 |
| Downregulated genes (alpha=5e-7) | *SCIMP* | 388325 | SLP adaptor and CSK interacting membrane protein |
| Downregulated genes (alpha=5e-7) | *SERPINA9* | 327657 | serpin family A member 9 |
| Downregulated genes (alpha=5e-7) | *SGPP2* | 130367 | sphingosine-1-phosphate phosphatase 2 |
| Downregulated genes (alpha=5e-7) | *SH2D2A* | 9047 | SH2 domain containing 2A |
| Downregulated genes (alpha=5e-7) | *SIT1* | 27240 | signaling threshold regulating transmembrane adaptor 1 |
| Downregulated genes (alpha=5e-7) | *SLAMF1* | 6504 | signaling lymphocytic activation molecule family member 1 |
| Downregulated genes (alpha=5e-7) | *SLC16A6* | 9120 | solute carrier family 16 member 6 |
| Downregulated genes (alpha=5e-7) | *SLC17A9* | 63910 | solute carrier family 17 member 9 |
| Downregulated genes (alpha=5e-7) | *SLC45A3* | 85414 | solute carrier family 45 member 3 |
| Downregulated genes (alpha=5e-7) | *SNX22* | 79856 | sorting nexin 22 |
| Downregulated genes (alpha=5e-7) | *SYNGR2* | 9144 | synaptogyrin 2 |
| Downregulated genes (alpha=5e-7) | *TLR10* | 81793 | toll like receptor 10 |
| Downregulated genes (alpha=5e-7) | *TMEM200A* | 114801 | transmembrane protein 200A |
| Downregulated genes (alpha=5e-7) | *TNF* | 7124 | tumor necrosis factor |
| Downregulated genes (alpha=5e-7) | *TUBB2A* | 7280 | tubulin beta 2A class IIa |
| Downregulated genes (alpha=5e-7) | *TUBB2B* | 347733 | tubulin beta 2B class IIb |
| Downregulated genes (alpha=5e-7) | *UPP1* | 7378 | uridine phosphorylase 1 |
| Downregulated genes (alpha=5e-7) | *VEGFA* | 7422 | vascular endothelial growth factor A |
| Downregulated genes (alpha=5e-7) | *VPREB3* | 29802 | V-set pre-B cell surrogate light chain 3 |
| Downregulated genes (alpha=5e-7) | *ZMIZ1* | 57178 | zinc finger MIZ-type containing 1 |
| Downregulated genes (alpha=5e-7) | *ZNF318* | 24149 | zinc finger protein 318 |
| Upregulated genes (alpha=e-15) | *ADA* | 100 | adenosine deaminase |
| Upregulated genes (alpha=e-15) | *AFF3* | 3899 | AF4/FMR2 family member 3 |
| Upregulated genes (alpha=e-15) | *AICDA* | 57379 | activation induced cytidine deaminase |
| Upregulated genes (alpha=e-15) | *ALDH5A1* | 7915 | aldehyde dehydrogenase 5 family member A1 |
| Upregulated genes (alpha=e-15) | *ANKRD36BP2* | 645784 | ankyrin repeat domain 36B pseudogene 2 |
| Upregulated genes (alpha=e-15) | *APOD* | 347 | apolipoprotein D |
| Upregulated genes (alpha=e-15) | *ARHGAP27P2* | 440461 | Rho GTPase activating protein 27 pseudogene 2 |
| Upregulated genes (alpha=e-15) | *ARID5B* | 84159 | AT-rich interaction domain 5B |
| Upregulated genes (alpha=e-15) | *BACH2* | 60468 | BTB domain and CNC homolog 2 |
| Upregulated genes (alpha=e-15) | *BBC3* | 27113 | BCL2 binding component 3 |
| Upregulated genes (alpha=e-15) | *BCL2L11* | 10018 | BCL2 like 11 |
| Upregulated genes (alpha=e-15) | *BCR* | 613 | BCR activator of RhoGEF and GTPase |
| Upregulated genes (alpha=e-15) | *BLOC1S6* | 26258 | biogenesis of lysosomal organelles complex 1 subunit 6 |
| Upregulated genes (alpha=e-15) | *BMP7* | 655 | bone morphogenetic protein 7 |
| Upregulated genes (alpha=e-15) | *BTG1* | 694 | BTG anti-proliferation factor 1 |
| Upregulated genes (alpha=e-15) | *C16orf54* | 283897 | chromosome 16 open reading frame 54 |
| Upregulated genes (alpha=e-15) | *CASTOR2* | 729438 | cytosolic arginine sensor for mTORC1 subunit 2 |
| Upregulated genes (alpha=e-15) | *CDK13* | 8621 | cyclin dependent kinase 13 |
| Upregulated genes (alpha=e-15) | *CEP128* | 145508 | centrosomal protein 128 |
| Upregulated genes (alpha=e-15) | *CHPT1* | 56994 | choline phosphotransferase 1 |
| Upregulated genes (alpha=e-15) | *CHST15* | 51363 | carbohydrate sulfotransferase 15 |
| Upregulated genes (alpha=e-15) | *CIPC* | 85457 | CLOCK interacting pacemaker |
| Upregulated genes (alpha=e-15) | *CSNK1G3* | 1456 | casein kinase 1 gamma 3 |
| Upregulated genes (alpha=e-15) | *CTDSP2* | 10106 | CTD small phosphatase 2 |
| Upregulated genes (alpha=e-15) | *CXCR4* | 7852 | C-X-C motif chemokine receptor 4 |
| Upregulated genes (alpha=e-15) | *DIP2C* | 22982 | disco interacting protein 2 homolog C |
| Upregulated genes (alpha=e-15) | *DNMT3B* | 1789 | DNA methyltransferase 3 beta |
| Upregulated genes (alpha=e-15) | *EIF2AK3* | 9451 | eukaryotic translation initiation factor 2 alpha kinase 3 |
| Upregulated genes (alpha=e-15) | *FBXO30* | 84085 | F-box protein 30 |
| Upregulated genes (alpha=e-15) | *FCGBP* | 8857 | Fc fragment of IgG binding protein |
| Upregulated genes (alpha=e-15) | *FOXL1* | 2300 | forkhead box L1 |
| Upregulated genes (alpha=e-15) | *FOXP1* | 27086 | forkhead box P1 |
| Upregulated genes (alpha=e-15) | *GCSAM* | 257144 | germinal center associated signaling and motility |
| Upregulated genes (alpha=e-15) | *GPLD1* | 2822 | glycosylphosphatidylinositol specific phospholipase D1 |
| Upregulated genes (alpha=e-15) | *GPM6A* | 2823 | glycoprotein M6A |
| Upregulated genes (alpha=e-15) | *HIPK2* | 28996 | homeodomain interacting protein kinase 2 |
| Upregulated genes (alpha=e-15) | *HRK* | 8739 | harakiri, BCL2 interacting protein |
| Upregulated genes (alpha=e-15) | *ITGB1* | 3688 | integrin subunit beta 1 |
| Upregulated genes (alpha=e-15) | *JAK1* | 3716 | Janus kinase 1 |
| Upregulated genes (alpha=e-15) | *KANK2* | 25959 | KN motif and ankyrin repeat domains 2 |
| Upregulated genes (alpha=e-15) | *KLHL14* | 57565 | kelch like family member 14 |
| Upregulated genes (alpha=e-15) | *LAMA5* | 3911 | laminin subunit alpha 5 |
| Upregulated genes (alpha=e-15) | *LDLRAD4* | 753 | low density lipoprotein receptor class A domain containing 4 |
| Upregulated genes (alpha=e-15) | *LOC101929322* | 101929322 | integrator complex subunit 4 pseudogene |
| Upregulated genes (alpha=e-15) | *LOC112268313* | 112268313 | collagen alpha-1(I) chain-like |
| Upregulated genes (alpha=e-15) | *LPP* | 4026 | LIM domain containing preferred translocation partner in lipoma |
| Upregulated genes (alpha=e-15) | *MAP3K1* | 4214 | mitogen-activated protein kinase kinase kinase 1 |
| Upregulated genes (alpha=e-15) | *MME* | 4311 | membrane metalloendopeptidase |
| Upregulated genes (alpha=e-15) | *NEIL1* | 79661 | nei like DNA glycosylase 1 |
| Upregulated genes (alpha=e-15) | *NUAK2* | 81788 | NUAK family kinase 2 |
| Upregulated genes (alpha=e-15) | *OGFRL1* | 79627 | opioid growth factor receptor like 1 |
| Upregulated genes (alpha=e-15) | *PIK3CA* | 5290 | phosphatidylinositol-4,5-bisphosphate 3-kinase catalytic subunit alpha |
| Upregulated genes (alpha=e-15) | *PIM1* | 5292 | Pim-1 proto-oncogene, serine/threonine kinase |
| Upregulated genes (alpha=e-15) | *PLEKHG1* | 57480 | pleckstrin homology and RhoGEF domain containing G1 |
| Upregulated genes (alpha=e-15) | *POU2AF1* | 5450 | POU class 2 homeobox associating factor 1 |
| Upregulated genes (alpha=e-15) | *PRDM15* | 63977 | PR/SET domain 15 |
| Upregulated genes (alpha=e-15) | *PRKCB* | 5579 | protein kinase C beta |
| Upregulated genes (alpha=e-15) | *PRKCE* | 5581 | protein kinase C epsilon |
| Upregulated genes (alpha=e-15) | *PYHIN1* | 149628 | pyrin and HIN domain family member 1 |
| Upregulated genes (alpha=e-15) | *RASSF6* | 166824 | Ras association domain family member 6 |
| Upregulated genes (alpha=e-15) | *RCSD1* | 92241 | RCSD domain containing 1 |
| Upregulated genes (alpha=e-15) | *REPIN1* | 29803 | replication initiator 1 |
| Upregulated genes (alpha=e-15) | *RIMS3* | 9783 | regulating synaptic membrane exocytosis 3 |
| Upregulated genes (alpha=e-15) | *RMI2* | 116028 | RecQ mediated genome instability 2 |
| Upregulated genes (alpha=e-15) | *RNA45SN1* | 106631777 | RNA, 45S pre-ribosomal N1 |
| Upregulated genes (alpha=e-15) | *RNA45SN2* | 109864279 | RNA, 45S pre-ribosomal N2 |
| Upregulated genes (alpha=e-15) | *RNA45SN3* | 109910379 | RNA, 45S pre-ribosomal N3 |
| Upregulated genes (alpha=e-15) | *RNA45SN4* | 109864271 | RNA, 45S pre-ribosomal N4 |
| Upregulated genes (alpha=e-15) | *RNF144B* | 255488 | ring finger protein 144B |
| Upregulated genes (alpha=e-15) | *RRM2B* | 50484 | ribonucleotide reductase regulatory TP53 inducible subunit M2B |
| Upregulated genes (alpha=e-15) | *RUBCNL* | 80183 | rubicon like autophagy enhancer |
| Upregulated genes (alpha=e-15) | *SAMD12* | 401474 | sterile alpha motif domain containing 12 |
| Upregulated genes (alpha=e-15) | *SAV1* | 60485 | salvador family WW domain containing protein 1 |
| Upregulated genes (alpha=e-15) | *SCN4A* | 6329 | sodium voltage-gated channel alpha subunit 4 |
| Upregulated genes (alpha=e-15) | *SH3TC1* | 54436 | SH3 domain and tetratricopeptide repeats 1 |
| Upregulated genes (alpha=e-15) | *SLAMF6* | 114836 | SLAM family member 6 |
| Upregulated genes (alpha=e-15) | *SLC30A4* | 7782 | solute carrier family 30 member 4 |
| Upregulated genes (alpha=e-15) | *SLC35E3* | 55508 | solute carrier family 35 member E3 |
| Upregulated genes (alpha=e-15) | *SLC4A10* | 57282 | solute carrier family 4 member 10 |
| Upregulated genes (alpha=e-15) | *SNTB1* | 6641 | syntrophin beta 1 |
| Upregulated genes (alpha=e-15) | *SOCS1* | 8651 | suppressor of cytokine signaling 1 |
| Upregulated genes (alpha=e-15) | *STAT2* | 6773 | signal transducer and activator of transcription 2 |
| Upregulated genes (alpha=e-15) | *SUSD3* | 203328 | sushi domain containing 3 |
| Upregulated genes (alpha=e-15) | *SYK* | 6850 | spleen associated tyrosine kinase |
| Upregulated genes (alpha=e-15) | *SYTL2* | 54843 | synaptotagmin like 2 |
| Upregulated genes (alpha=e-15) | *TAGAP* | 117289 | T cell activation RhoGTPase activating protein |
| Upregulated genes (alpha=e-15) | *TNFSF8* | 944 | TNF superfamily member 8 |
| Upregulated genes (alpha=e-15) | *TP53INP1* | 94241 | tumor protein p53 inducible nuclear protein 1 |
| Upregulated genes (alpha=e-15) | *TP63* | 8626 | tumor protein p63 |
| Upregulated genes (alpha=e-15) | *TTC28* | 23331 | tetratricopeptide repeat domain 28 |
| Upregulated genes (alpha=e-15) | *WNK2* | 65268 | WNK lysine deficient protein kinase 2 |
| Upregulated genes (alpha=e-15) | *YPEL3* | 83719 | yippee like 3 |
| Upregulated genes (alpha=e-15) | *ZBTB18* | 10472 | zinc finger and BTB domain containing 18 |
| Upregulated genes (alpha=e-15) | *ZDBF2* | 57683 | zinc finger DBF-type containing 2 |
| Upregulated genes (alpha=e-15) | *ZEB2* | 9839 | zinc finger E-box binding homeobox 2 |
| Upregulated genes (alpha=e-15) | *ZNF385B* | 151126 | zinc finger protein 385B |
| Upregulated genes (alpha=e-15) | *ZNF521* | 25925 | zinc finger protein 521 |

**Supplementary Table S4**

| **Top genes after the treatment of 5 nM rituximab in SU-DHL-6** | | | |
| --- | --- | --- | --- |
| Signature name | Gene symbol | GeneID | Gene description |
| Downregulated genes (alpha=5e-7) | *ABI3* | 51225 | ABI family member 3 |
| Downregulated genes (alpha=5e-7) | *ACY3* | 91703 | aminoacylase 3 |
| Downregulated genes (alpha=5e-7) | *ALPL* | 249 | alkaline phosphatase, biomineralization associated |
| Downregulated genes (alpha=5e-7) | *ASPH* | 444 | aspartate beta-hydroxylase |
| Downregulated genes (alpha=5e-7) | *C12orf75* | 387882 | chromosome 12 open reading frame 75 |
| Downregulated genes (alpha=5e-7) | *CAMK1D* | 57118 | calcium/calmodulin dependent protein kinase ID |
| Downregulated genes (alpha=5e-7) | *CBFA2T3* | 863 | CBFA2/RUNX1 partner transcriptional co-repressor 3 |
| Downregulated genes (alpha=5e-7) | *CCDC28B* | 79140 | coiled-coil domain containing 28B |
| Downregulated genes (alpha=5e-7) | *CCND3* | 896 | cyclin D3 |
| Downregulated genes (alpha=5e-7) | *CCNE2* | 9134 | cyclin E2 |
| Downregulated genes (alpha=5e-7) | *CFAP251* | 144406 | cilia and flagella associated protein 251 |
| Downregulated genes (alpha=5e-7) | *CHDH* | 55349 | choline dehydrogenase |
| Downregulated genes (alpha=5e-7) | *CISD3* | 284106 | CDGSH iron sulfur domain 3 |
| Downregulated genes (alpha=5e-7) | *CUX2* | 23316 | cut like homeobox 2 |
| Downregulated genes (alpha=5e-7) | *DDN* | 23109 | dendrin |
| Downregulated genes (alpha=5e-7) | *E2F1* | 1869 | E2F transcription factor 1 |
| Downregulated genes (alpha=5e-7) | *E2F2* | 1870 | E2F transcription factor 2 |
| Downregulated genes (alpha=5e-7) | *ELL3* | 80237 | elongation factor for RNA polymerase II 3 |
| Downregulated genes (alpha=5e-7) | *ESPNL* | 339768 | espin like |
| Downregulated genes (alpha=5e-7) | *GCNT2* | 2651 | glucosaminyl (N-acetyl) transferase 2 (I blood group) |
| Downregulated genes (alpha=5e-7) | *GRAP* | 10750 | GRB2 related adaptor protein |
| Downregulated genes (alpha=5e-7) | *GRAPL* | 400581 | GRB2 related adaptor protein like |
| Downregulated genes (alpha=5e-7) | *HYPK* | 25764 | huntingtin interacting protein K |
| Downregulated genes (alpha=5e-7) | *ICAM1* | 3383 | intercellular adhesion molecule 1 |
| Downregulated genes (alpha=5e-7) | *ID1* | 3397 | inhibitor of DNA binding 1, HLH protein |
| Downregulated genes (alpha=5e-7) | *IL17RB* | 55540 | interleukin 17 receptor B |
| Downregulated genes (alpha=5e-7) | *ITGB7* | 3695 | integrin subunit beta 7 |
| Downregulated genes (alpha=5e-7) | *LAT2* | 7462 | linker for activation of T cells family member 2 |
| Downregulated genes (alpha=5e-7) | *LIME1* | 54923 | Lck interacting transmembrane adaptor 1 |
| Downregulated genes (alpha=5e-7) | *LY86* | 9450 | lymphocyte antigen 86 |
| Downregulated genes (alpha=5e-7) | *MGAT3* | 4248 | beta-1,4-mannosyl-glycoprotein 4-beta-N-acetylglucosaminyltransferase |
| Downregulated genes (alpha=5e-7) | *MYADML2* | 255275 | myeloid associated differentiation marker like 2 |
| Downregulated genes (alpha=5e-7) | *MYH11* | 4629 | myosin heavy chain 11 |
| Downregulated genes (alpha=5e-7) | *NCF1* | 653361 | neutrophil cytosolic factor 1 |
| Downregulated genes (alpha=5e-7) | *NCF1B* | 654816 | neutrophil cytosolic factor 1B pseudogene |
| Downregulated genes (alpha=5e-7) | *NCF1C* | 654817 | neutrophil cytosolic factor 1C pseudogene |
| Downregulated genes (alpha=5e-7) | *NECTIN1* | 5818 | nectin cell adhesion molecule 1 |
| Downregulated genes (alpha=5e-7) | *NLRC3* | 197358 | NLR family CARD domain containing 3 |
| Downregulated genes (alpha=5e-7) | *NLRP11* | 204801 | NLR family pyrin domain containing 11 |
| Downregulated genes (alpha=5e-7) | *NLRP7* | 199713 | NLR family pyrin domain containing 7 |
| Downregulated genes (alpha=5e-7) | *NOTUM* | 147111 | notum, palmitoleoyl-protein carboxylesterase |
| Downregulated genes (alpha=5e-7) | *NSG1* | 27065 | neuronal vesicle trafficking associated 1 |
| Downregulated genes (alpha=5e-7) | *NXPH4* | 11247 | neurexophilin 4 |
| Downregulated genes (alpha=5e-7) | *PACSIN1* | 29993 | protein kinase C and casein kinase substrate in neurons 1 |
| Downregulated genes (alpha=5e-7) | *PBK* | 55872 | PDZ binding kinase |
| Downregulated genes (alpha=5e-7) | *PDZRN3* | 23024 | PDZ domain containing ring finger 3 |
| Downregulated genes (alpha=5e-7) | *PITX1* | 5307 | paired like homeodomain 1 |
| Downregulated genes (alpha=5e-7) | *PLD4* | 122618 | phospholipase D family member 4 |
| Downregulated genes (alpha=5e-7) | *PLEKHA4* | 57664 | pleckstrin homology domain containing A4 |
| Downregulated genes (alpha=5e-7) | *POLR1G* | 10849 | RNA polymerase I subunit G |
| Downregulated genes (alpha=5e-7) | *PRDM10* | 56980 | PR/SET domain 10 |
| Downregulated genes (alpha=5e-7) | *PVRIG* | 79037 | PVR related immunoglobulin domain containing |
| Downregulated genes (alpha=5e-7) | *SERPINA9* | 327657 | serpin family A member 9 |
| Downregulated genes (alpha=5e-7) | *SLC45A3* | 85414 | solute carrier family 45 member 3 |
| Downregulated genes (alpha=5e-7) | *SNX22* | 79856 | sorting nexin 22 |
| Downregulated genes (alpha=5e-7) | *SSBP2* | 23635 | single stranded DNA binding protein 2 |
| Downregulated genes (alpha=5e-7) | *TAF4B* | 6875 | TATA-box binding protein associated factor 4b |
| Downregulated genes (alpha=5e-7) | *TCL1A* | 8115 | TCL1 family AKT coactivator A |
| Downregulated genes (alpha=5e-7) | *TCL1B* | 9623 | TCL1 family AKT coactivator B |
| Downregulated genes (alpha=5e-7) | *TERT* | 7015 | telomerase reverse transcriptase |
| Downregulated genes (alpha=5e-7) | *TFAP4* | 7023 | transcription factor AP-4 |
| Downregulated genes (alpha=5e-7) | *TMEM229B* | 161145 | transmembrane protein 229B |
| Downregulated genes (alpha=5e-7) | *TUBB3* | 10381 | tubulin beta 3 class III |
| Downregulated genes (alpha=5e-7) | *VPREB3* | 29802 | V-set pre-B cell surrogate light chain 3 |
| Downregulated genes (alpha=5e-7) | *ZNF318* | 24149 | zinc finger protein 318 |
| Upregulated genes (alpha=e-15) | *ABAT* | 18 | 4-aminobutyrate aminotransferase |
| Upregulated genes (alpha=e-15) | *AHNAK* | 79026 | AHNAK nucleoprotein |
| Upregulated genes (alpha=e-15) | *AICDA* | 57379 | activation induced cytidine deaminase |
| Upregulated genes (alpha=e-15) | *AMIGO2* | 347902 | adhesion molecule with Ig like domain 2 |
| Upregulated genes (alpha=e-15) | *APOD* | 347 | apolipoprotein D |
| Upregulated genes (alpha=e-15) | *ARID5B* | 84159 | AT-rich interaction domain 5B |
| Upregulated genes (alpha=e-15) | *ATXN1* | 6310 | ataxin 1 |
| Upregulated genes (alpha=e-15) | *BACH2* | 60468 | BTB domain and CNC homolog 2 |
| Upregulated genes (alpha=e-15) | *BCL2A1* | 597 | BCL2 related protein A1 |
| Upregulated genes (alpha=e-15) | *BCL2L11* | 10018 | BCL2 like 11 |
| Upregulated genes (alpha=e-15) | *BIK* | 638 | BCL2 interacting killer |
| Upregulated genes (alpha=e-15) | *C16orf74* | 404550 | chromosome 16 open reading frame 74 |
| Upregulated genes (alpha=e-15) | *CD44* | 960 | CD44 molecule (Indian blood group) |
| Upregulated genes (alpha=e-15) | *CD83* | 9308 | CD83 molecule |
| Upregulated genes (alpha=e-15) | *CDHR3* | 222256 | cadherin related family member 3 |
| Upregulated genes (alpha=e-15) | *CELF2* | 10659 | CUGBP Elav-like family member 2 |
| Upregulated genes (alpha=e-15) | *CHST2* | 9435 | carbohydrate sulfotransferase 2 |
| Upregulated genes (alpha=e-15) | *CIITA* | 4261 | class II major histocompatibility complex transactivator |
| Upregulated genes (alpha=e-15) | *CLEC17A* | 388512 | C-type lectin domain containing 17A |
| Upregulated genes (alpha=e-15) | *COBLL1* | 22837 | cordon-bleu WH2 repeat protein like 1 |
| Upregulated genes (alpha=e-15) | *CSRNP1* | 64651 | cysteine and serine rich nuclear protein 1 |
| Upregulated genes (alpha=e-15) | *CTNNA1* | 1495 | catenin alpha 1 |
| Upregulated genes (alpha=e-15) | *DUSP10* | 11221 | dual specificity phosphatase 10 |
| Upregulated genes (alpha=e-15) | *DUSP2* | 1844 | dual specificity phosphatase 2 |
| Upregulated genes (alpha=e-15) | *DUSP5* | 1847 | dual specificity phosphatase 5 |
| Upregulated genes (alpha=e-15) | *EGR2* | 1959 | early growth response 2 |
| Upregulated genes (alpha=e-15) | *EGR3* | 1960 | early growth response 3 |
| Upregulated genes (alpha=e-15) | *EVI2A* | 2123 | ecotropic viral integration site 2A |
| Upregulated genes (alpha=e-15) | *EVI2B* | 2124 | ecotropic viral integration site 2B |
| Upregulated genes (alpha=e-15) | *FAM3C* | 10447 | FAM3 metabolism regulating signaling molecule C |
| Upregulated genes (alpha=e-15) | *FBXO30* | 84085 | F-box protein 30 |
| Upregulated genes (alpha=e-15) | *FCRL5* | 83416 | Fc receptor like 5 |
| Upregulated genes (alpha=e-15) | *FOXP1* | 27086 | forkhead box P1 |
| Upregulated genes (alpha=e-15) | *FURIN* | 5045 | furin, paired basic amino acid cleaving enzyme |
| Upregulated genes (alpha=e-15) | *FYN* | 2534 | FYN proto-oncogene, Src family tyrosine kinase |
| Upregulated genes (alpha=e-15) | *GAS7* | 8522 | growth arrest specific 7 |
| Upregulated genes (alpha=e-15) | *GFOD1* | 54438 | glucose-fructose oxidoreductase domain containing 1 |
| Upregulated genes (alpha=e-15) | *HIC1* | 3090 | HIC ZBTB transcriptional repressor 1 |
| Upregulated genes (alpha=e-15) | *HIVEP3* | 59269 | HIVEP zinc finger 3 |
| Upregulated genes (alpha=e-15) | *HLA-DMA* | 3108 | major histocompatibility complex, class II, DM alpha |
| Upregulated genes (alpha=e-15) | *HLA-DQB1* | 3119 | major histocompatibility complex, class II, DQ beta 1 |
| Upregulated genes (alpha=e-15) | *HLA-DQB2* | 3120 | major histocompatibility complex, class II, DQ beta 2 |
| Upregulated genes (alpha=e-15) | *HLA-DRB6* | 3128 | major histocompatibility complex, class II, DR beta 6 (pseudogene) |
| Upregulated genes (alpha=e-15) | *HMOX1* | 3162 | heme oxygenase 1 |
| Upregulated genes (alpha=e-15) | *HOMER2* | 9455 | homer scaffold protein 2 |
| Upregulated genes (alpha=e-15) | *IKZF2* | 22807 | IKAROS family zinc finger 2 |
| Upregulated genes (alpha=e-15) | *IL16* | 3603 | interleukin 16 |
| Upregulated genes (alpha=e-15) | *IL21R* | 50615 | interleukin 21 receptor |
| Upregulated genes (alpha=e-15) | *IL4I1* | 259307 | interleukin 4 induced 1 |
| Upregulated genes (alpha=e-15) | *INPP5F* | 22876 | inositol polyphosphate-5-phosphatase F |
| Upregulated genes (alpha=e-15) | *IRF5* | 3663 | interferon regulatory factor 5 |
| Upregulated genes (alpha=e-15) | *ITPRIPL2* | 162073 | ITPRIP like 2 |
| Upregulated genes (alpha=e-15) | *JSRP1* | 126306 | junctional sarcoplasmic reticulum protein 1 |
| Upregulated genes (alpha=e-15) | *JUND* | 3727 | JunD proto-oncogene, AP-1 transcription factor subunit |
| Upregulated genes (alpha=e-15) | *KIF26B* | 55083 | kinesin family member 26B |
| Upregulated genes (alpha=e-15) | *LDLRAD4* | 753 | low density lipoprotein receptor class A domain containing 4 |
| Upregulated genes (alpha=e-15) | *LITAF* | 9516 | lipopolysaccharide induced TNF factor |
| Upregulated genes (alpha=e-15) | *LRIG1* | 26018 | leucine rich repeats and immunoglobulin like domains 1 |
| Upregulated genes (alpha=e-15) | *LRRK2* | 120892 | leucine rich repeat kinase 2 |
| Upregulated genes (alpha=e-15) | *LY9* | 4063 | lymphocyte antigen 9 |
| Upregulated genes (alpha=e-15) | *MAN1A1* | 4121 | mannosidase alpha class 1A member 1 |
| Upregulated genes (alpha=e-15) | *MAP3K1* | 4214 | mitogen-activated protein kinase kinase kinase 1 |
| Upregulated genes (alpha=e-15) | *MDFIC* | 29969 | MyoD family inhibitor domain containing |
| Upregulated genes (alpha=e-15) | *MPEG1* | 219972 | macrophage expressed 1 |
| Upregulated genes (alpha=e-15) | *NAB2* | 4665 | NGFI-A binding protein 2 |
| Upregulated genes (alpha=e-15) | *NFATC1* | 4772 | nuclear factor of activated T cells 1 |
| Upregulated genes (alpha=e-15) | *NR4A1* | 3164 | nuclear receptor subfamily 4 group A member 1 |
| Upregulated genes (alpha=e-15) | *NSMCE3* | 56160 | NSE3 homolog, SMC5-SMC6 complex component |
| Upregulated genes (alpha=e-15) | *P2RY10* | 27334 | P2Y receptor family member 10 |
| Upregulated genes (alpha=e-15) | *PCDH9* | 5101 | protocadherin 9 |
| Upregulated genes (alpha=e-15) | *PHACTR1* | 221692 | phosphatase and actin regulator 1 |
| Upregulated genes (alpha=e-15) | *PHLDA1* | 22822 | pleckstrin homology like domain family A member 1 |
| Upregulated genes (alpha=e-15) | *PLXNC1* | 10154 | plexin C1 |
| Upregulated genes (alpha=e-15) | *PTPN6* | 5777 | protein tyrosine phosphatase non-receptor type 6 |
| Upregulated genes (alpha=e-15) | *PYHIN1* | 149628 | pyrin and HIN domain family member 1 |
| Upregulated genes (alpha=e-15) | *RASGEF1B* | 153020 | RasGEF domain family member 1B |
| Upregulated genes (alpha=e-15) | *RASSF6* | 166824 | Ras association domain family member 6 |
| Upregulated genes (alpha=e-15) | *REPIN1* | 29803 | replication initiator 1 |
| Upregulated genes (alpha=e-15) | *RGS3* | 5998 | regulator of G protein signaling 3 |
| Upregulated genes (alpha=e-15) | *RN7SK* | 125050 | RNA component of 7SK nuclear ribonucleoprotein |
| Upregulated genes (alpha=e-15) | *RNA45SN1* | 106631777 | RNA, 45S pre-ribosomal N1 |
| Upregulated genes (alpha=e-15) | *RNA45SN2* | 109864279 | RNA, 45S pre-ribosomal N2 |
| Upregulated genes (alpha=e-15) | *RNA45SN3* | 109910379 | RNA, 45S pre-ribosomal N3 |
| Upregulated genes (alpha=e-15) | *RNA45SN4* | 109864271 | RNA, 45S pre-ribosomal N4 |
| Upregulated genes (alpha=e-15) | *SCIMP* | 388325 | SLP adaptor and CSK interacting membrane protein |
| Upregulated genes (alpha=e-15) | *SEMA7A* | 8482 | semaphorin 7A (John Milton Hagen blood group) |
| Upregulated genes (alpha=e-15) | *SH2D2A* | 9047 | SH2 domain containing 2A |
| Upregulated genes (alpha=e-15) | *SH3BP5* | 9467 | SH3 domain binding protein 5 |
| Upregulated genes (alpha=e-15) | *SLAMF7* | 57823 | SLAM family member 7 |
| Upregulated genes (alpha=e-15) | *SLC44A2* | 57153 | solute carrier family 44 member 2 |
| Upregulated genes (alpha=e-15) | *SNX9* | 51429 | sorting nexin 9 |
| Upregulated genes (alpha=e-15) | *ST3GAL1* | 6482 | ST3 beta-galactoside alpha-2,3-sialyltransferase 1 |
| Upregulated genes (alpha=e-15) | *SYT17* | 51760 | synaptotagmin 17 |
| Upregulated genes (alpha=e-15) | *TAGAP* | 117289 | T cell activation RhoGTPase activating protein |
| Upregulated genes (alpha=e-15) | *TNFRSF18* | 8784 | TNF receptor superfamily member 18 |
| Upregulated genes (alpha=e-15) | *TNFRSF1B* | 7133 | TNF receptor superfamily member 1B |
| Upregulated genes (alpha=e-15) | *TP53INP1* | 94241 | tumor protein p53 inducible nuclear protein 1 |
| Upregulated genes (alpha=e-15) | *TRAF1* | 7185 | TNF receptor associated factor 1 |
| Upregulated genes (alpha=e-15) | *TRIB1* | 10221 | tribbles pseudokinase 1 |
| Upregulated genes (alpha=e-15) | *TSC22D3* | 1831 | TSC22 domain family member 3 |
| Upregulated genes (alpha=e-15) | *TTYH3* | 80727 | tweety family member 3 |
| Upregulated genes (alpha=e-15) | *UGCG* | 7357 | UDP-glucose ceramide glucosyltransferase |
| Upregulated genes (alpha=e-15) | *VAV3* | 10451 | vav guanine nucleotide exchange factor 3 |
| Upregulated genes (alpha=e-15) | *ZDBF2* | 57683 | zinc finger DBF-type containing 2 |
| Upregulated genes (alpha=e-15) | *ZEB2* | 9839 | zinc finger E-box binding homeobox 2 |
| Upregulated genes (alpha=e-15) | *ZFP36L1* | 677 | ZFP36 ring finger protein like 1 |
| Upregulated genes (alpha=e-15) | *ZYX* | 7791 | zyxin |

**Supplementary Table S5**

| **Top genes after the combination treatment of 5 nM tafasitamab and 5 nM rituximab in SU-DHL-6** | | | |
| --- | --- | --- | --- |
| Signature name | Gene symbol | GeneID | Gene description |
| Downregulated genes (alpha=5e-7) | *ABI3* | 51225 | ABI family member 3 |
| Downregulated genes (alpha=5e-7) | *ACY3* | 91703 | aminoacylase 3 |
| Downregulated genes (alpha=5e-7) | *ALPL* | 249 | alkaline phosphatase, biomineralization associated |
| Downregulated genes (alpha=5e-7) | *ASPH* | 444 | aspartate beta-hydroxylase |
| Downregulated genes (alpha=5e-7) | *ATF5* | 22809 | activating transcription factor 5 |
| Downregulated genes (alpha=5e-7) | *BPNT1* | 10380 | 3'(2'), 5'-bisphosphate nucleotidase 1 |
| Downregulated genes (alpha=5e-7) | *C12orf75* | 387882 | chromosome 12 open reading frame 75 |
| Downregulated genes (alpha=5e-7) | *CAMP* | 820 | cathelicidin antimicrobial peptide |
| Downregulated genes (alpha=5e-7) | *CBFA2T3* | 863 | CBFA2/RUNX1 partner transcriptional co-repressor 3 |
| Downregulated genes (alpha=5e-7) | *CBS* | 875 | cystathionine beta-synthase |
| Downregulated genes (alpha=5e-7) | *CCDC110* | 256309 | coiled-coil domain containing 110 |
| Downregulated genes (alpha=5e-7) | *CCNE2* | 9134 | cyclin E2 |
| Downregulated genes (alpha=5e-7) | *CDC25A* | 993 | cell division cycle 25A |
| Downregulated genes (alpha=5e-7) | *CHCHD10* | 400916 | coiled-coil-helix-coiled-coil-helix domain containing 10 |
| Downregulated genes (alpha=5e-7) | *CHDH* | 55349 | choline dehydrogenase |
| Downregulated genes (alpha=5e-7) | *CUX2* | 23316 | cut like homeobox 2 |
| Downregulated genes (alpha=5e-7) | *DDIT4* | 54541 | DNA damage inducible transcript 4 |
| Downregulated genes (alpha=5e-7) | *DDN* | 23109 | dendrin |
| Downregulated genes (alpha=5e-7) | *DENND3* | 22898 | DENN domain containing 3 |
| Downregulated genes (alpha=5e-7) | *E2F2* | 1870 | E2F transcription factor 2 |
| Downregulated genes (alpha=5e-7) | *ELL3* | 80237 | elongation factor for RNA polymerase II 3 |
| Downregulated genes (alpha=5e-7) | *ESPNL* | 339768 | espin like |
| Downregulated genes (alpha=5e-7) | *FKBP4* | 2288 | FKBP prolyl isomerase 4 |
| Downregulated genes (alpha=5e-7) | *GCNT2* | 2651 | glucosaminyl (N-acetyl) transferase 2 (I blood group) |
| Downregulated genes (alpha=5e-7) | *GRAP* | 10750 | GRB2 related adaptor protein |
| Downregulated genes (alpha=5e-7) | *HES1* | 3280 | hes family bHLH transcription factor 1 |
| Downregulated genes (alpha=5e-7) | *HSPA8* | 3312 | heat shock protein family A (Hsp70) member 8 |
| Downregulated genes (alpha=5e-7) | *HSPE1* | 3336 | heat shock protein family E (Hsp10) member 1 |
| Downregulated genes (alpha=5e-7) | *HTR3A* | 3359 | 5-hydroxytryptamine receptor 3A |
| Downregulated genes (alpha=5e-7) | *HYPK* | 25764 | huntingtin interacting protein K |
| Downregulated genes (alpha=5e-7) | *ICAM1* | 3383 | intercellular adhesion molecule 1 |
| Downregulated genes (alpha=5e-7) | *ID1* | 3397 | inhibitor of DNA binding 1, HLH protein |
| Downregulated genes (alpha=5e-7) | *IDH3A* | 3419 | isocitrate dehydrogenase (NAD(+)) 3 catalytic subunit alpha |
| Downregulated genes (alpha=5e-7) | *IFI30* | 10437 | IFI30 lysosomal thiol reductase |
| Downregulated genes (alpha=5e-7) | *IL17RB* | 55540 | interleukin 17 receptor B |
| Downregulated genes (alpha=5e-7) | *IQSEC1* | 9922 | IQ motif and Sec7 domain ArfGEF 1 |
| Downregulated genes (alpha=5e-7) | *ITGB7* | 3695 | integrin subunit beta 7 |
| Downregulated genes (alpha=5e-7) | *KCNK12* | 56660 | potassium two pore domain channel subfamily K member 12 |
| Downregulated genes (alpha=5e-7) | *LAT2* | 7462 | linker for activation of T cells family member 2 |
| Downregulated genes (alpha=5e-7) | *LIME1* | 54923 | Lck interacting transmembrane adaptor 1 |
| Downregulated genes (alpha=5e-7) | *LPCAT1* | 79888 | lysophosphatidylcholine acyltransferase 1 |
| Downregulated genes (alpha=5e-7) | *LY86* | 9450 | lymphocyte antigen 86 |
| Downregulated genes (alpha=5e-7) | *LYAR* | 55646 | Ly1 antibody reactive |
| Downregulated genes (alpha=5e-7) | *MACC1* | 346389 | MET transcriptional regulator MACC1 |
| Downregulated genes (alpha=5e-7) | *MRPL36* | 64979 | mitochondrial ribosomal protein L36 |
| Downregulated genes (alpha=5e-7) | *MYADML2* | 255275 | myeloid associated differentiation marker like 2 |
| Downregulated genes (alpha=5e-7) | *MYH11* | 4629 | myosin heavy chain 11 |
| Downregulated genes (alpha=5e-7) | *MZB1* | 51237 | marginal zone B and B1 cell specific protein |
| Downregulated genes (alpha=5e-7) | *NAPSB* | 256236 | napsin B aspartic peptidase, pseudogene |
| Downregulated genes (alpha=5e-7) | *NCF1* | 653361 | neutrophil cytosolic factor 1 |
| Downregulated genes (alpha=5e-7) | *NCF1C* | 654817 | neutrophil cytosolic factor 1C pseudogene |
| Downregulated genes (alpha=5e-7) | *NCR2* | 9436 | natural cytotoxicity triggering receptor 2 |
| Downregulated genes (alpha=5e-7) | *NLRP7* | 199713 | NLR family pyrin domain containing 7 |
| Downregulated genes (alpha=5e-7) | *NOTUM* | 147111 | notum, palmitoleoyl-protein carboxylesterase |
| Downregulated genes (alpha=5e-7) | *NXPH4* | 11247 | neurexophilin 4 |
| Downregulated genes (alpha=5e-7) | *PACSIN1* | 29993 | protein kinase C and casein kinase substrate in neurons 1 |
| Downregulated genes (alpha=5e-7) | *PAK1IP1* | 55003 | PAK1 interacting protein 1 |
| Downregulated genes (alpha=5e-7) | *PCDHGC3* | 5098 | protocadherin gamma subfamily C, 3 |
| Downregulated genes (alpha=5e-7) | *PDE9A* | 5152 | phosphodiesterase 9A |
| Downregulated genes (alpha=5e-7) | *PDZRN3* | 23024 | PDZ domain containing ring finger 3 |
| Downregulated genes (alpha=5e-7) | *PITX1* | 5307 | paired like homeodomain 1 |
| Downregulated genes (alpha=5e-7) | *PLD4* | 122618 | phospholipase D family member 4 |
| Downregulated genes (alpha=5e-7) | *PLEKHA4* | 57664 | pleckstrin homology domain containing A4 |
| Downregulated genes (alpha=5e-7) | *POLR1G* | 10849 | RNA polymerase I subunit G |
| Downregulated genes (alpha=5e-7) | *POLR3G* | 10622 | RNA polymerase III subunit G |
| Downregulated genes (alpha=5e-7) | *PRKCD* | 5580 | protein kinase C delta |
| Downregulated genes (alpha=5e-7) | *PVRIG* | 79037 | PVR related immunoglobulin domain containing |
| Downregulated genes (alpha=5e-7) | *PYCR1* | 5831 | pyrroline-5-carboxylate reductase 1 |
| Downregulated genes (alpha=5e-7) | *RABEPK* | 10244 | Rab9 effector protein with kelch motifs |
| Downregulated genes (alpha=5e-7) | *RAP1GAP2* | 23108 | RAP1 GTPase activating protein 2 |
| Downregulated genes (alpha=5e-7) | *RCAN2* | 10231 | regulator of calcineurin 2 |
| Downregulated genes (alpha=5e-7) | *RGS16* | 6004 | regulator of G protein signaling 16 |
| Downregulated genes (alpha=5e-7) | *SCARB1* | 949 | scavenger receptor class B member 1 |
| Downregulated genes (alpha=5e-7) | *SCNN1G* | 6340 | sodium channel epithelial 1 subunit gamma |
| Downregulated genes (alpha=5e-7) | *SERPINA9* | 327657 | serpin family A member 9 |
| Downregulated genes (alpha=5e-7) | *SH2B2* | 10603 | SH2B adaptor protein 2 |
| Downregulated genes (alpha=5e-7) | *SIT1* | 27240 | signaling threshold regulating transmembrane adaptor 1 |
| Downregulated genes (alpha=5e-7) | *SLC16A6* | 9120 | solute carrier family 16 member 6 |
| Downregulated genes (alpha=5e-7) | *SLC17A9* | 63910 | solute carrier family 17 member 9 |
| Downregulated genes (alpha=5e-7) | *SLC29A1* | 2030 | solute carrier family 29 member 1 (Augustine blood group) |
| Downregulated genes (alpha=5e-7) | *SLC45A3* | 85414 | solute carrier family 45 member 3 |
| Downregulated genes (alpha=5e-7) | *SNX22* | 79856 | sorting nexin 22 |
| Downregulated genes (alpha=5e-7) | *ST6GALNAC4* | 27090 | ST6 N-acetylgalactosaminide alpha-2,6-sialyltransferase 4 |
| Downregulated genes (alpha=5e-7) | *TERT* | 7015 | telomerase reverse transcriptase |
| Downregulated genes (alpha=5e-7) | *TLR10* | 81793 | toll like receptor 10 |
| Downregulated genes (alpha=5e-7) | *TMEM200A* | 114801 | transmembrane protein 200A |
| Downregulated genes (alpha=5e-7) | *TMEM229B* | 161145 | transmembrane protein 229B |
| Downregulated genes (alpha=5e-7) | *TRAF4* | 9618 | TNF receptor associated factor 4 |
| Downregulated genes (alpha=5e-7) | *TUBB2A* | 7280 | tubulin beta 2A class IIa |
| Downregulated genes (alpha=5e-7) | *TUBB2B* | 347733 | tubulin beta 2B class IIb |
| Downregulated genes (alpha=5e-7) | *TUBB3* | 10381 | tubulin beta 3 class III |
| Downregulated genes (alpha=5e-7) | *VEGFA* | 7422 | vascular endothelial growth factor A |
| Downregulated genes (alpha=5e-7) | *VPREB3* | 29802 | V-set pre-B cell surrogate light chain 3 |
| Downregulated genes (alpha=5e-7) | *WNT10A* | 80326 | Wnt family member 10A |
| Downregulated genes (alpha=5e-7) | *ZMIZ1* | 57178 | zinc finger MIZ-type containing 1 |
| Downregulated genes (alpha=5e-7) | *ZNF318* | 24149 | zinc finger protein 318 |
| Upregulated genes (alpha=e-15) | *ABLIM1* | 3983 | actin binding LIM protein 1 |
| Upregulated genes (alpha=e-15) | *ABTB1* | 80325 | ankyrin repeat and BTB domain containing 1 |
| Upregulated genes (alpha=e-15) | *ADA* | 100 | adenosine deaminase |
| Upregulated genes (alpha=e-15) | *AHNAK* | 79026 | AHNAK nucleoprotein |
| Upregulated genes (alpha=e-15) | *AICDA* | 57379 | activation induced cytidine deaminase |
| Upregulated genes (alpha=e-15) | *ALPK1* | 80216 | alpha kinase 1 |
| Upregulated genes (alpha=e-15) | *APOD* | 347 | apolipoprotein D |
| Upregulated genes (alpha=e-15) | *ARID5B* | 84159 | AT-rich interaction domain 5B |
| Upregulated genes (alpha=e-15) | *B3GALT4* | 8705 | beta-1,3-galactosyltransferase 4 |
| Upregulated genes (alpha=e-15) | *BACH2* | 60468 | BTB domain and CNC homolog 2 |
| Upregulated genes (alpha=e-15) | *BBC3* | 27113 | BCL2 binding component 3 |
| Upregulated genes (alpha=e-15) | *BCL2L11* | 10018 | BCL2 like 11 |
| Upregulated genes (alpha=e-15) | *BCR* | 613 | BCR activator of RhoGEF and GTPase |
| Upregulated genes (alpha=e-15) | *BLOC1S6* | 26258 | biogenesis of lysosomal organelles complex 1 subunit 6 |
| Upregulated genes (alpha=e-15) | *BTG1* | 694 | BTG anti-proliferation factor 1 |
| Upregulated genes (alpha=e-15) | *C16orf54* | 283897 | chromosome 16 open reading frame 54 |
| Upregulated genes (alpha=e-15) | *CASTOR2* | 729438 | cytosolic arginine sensor for mTORC1 subunit 2 |
| Upregulated genes (alpha=e-15) | *CD44* | 960 | CD44 molecule (Indian blood group) |
| Upregulated genes (alpha=e-15) | *CDK13* | 8621 | cyclin dependent kinase 13 |
| Upregulated genes (alpha=e-15) | *CEMIP2* | 23670 | cell migration inducing hyaluronidase 2 |
| Upregulated genes (alpha=e-15) | *CHST15* | 51363 | carbohydrate sulfotransferase 15 |
| Upregulated genes (alpha=e-15) | *CNR1* | 1268 | cannabinoid receptor 1 |
| Upregulated genes (alpha=e-15) | *COL9A3* | 1299 | collagen type IX alpha 3 chain |
| Upregulated genes (alpha=e-15) | *CPNE2* | 221184 | copine 2 |
| Upregulated genes (alpha=e-15) | *CSRNP1* | 64651 | cysteine and serine rich nuclear protein 1 |
| Upregulated genes (alpha=e-15) | *CTDSP2* | 10106 | CTD small phosphatase 2 |
| Upregulated genes (alpha=e-15) | *CXCR4* | 7852 | C-X-C motif chemokine receptor 4 |
| Upregulated genes (alpha=e-15) | *CYTIP* | 9595 | cytohesin 1 interacting protein |
| Upregulated genes (alpha=e-15) | *DIP2C* | 22982 | disco interacting protein 2 homolog C |
| Upregulated genes (alpha=e-15) | *EGR2* | 1959 | early growth response 2 |
| Upregulated genes (alpha=e-15) | *EIF2AK3* | 9451 | eukaryotic translation initiation factor 2 alpha kinase 3 |
| Upregulated genes (alpha=e-15) | *ELAPOR1* | 57535 | endosome-lysosome associated apoptosis and autophagy regulator 1 |
| Upregulated genes (alpha=e-15) | *EVI2A* | 2123 | ecotropic viral integration site 2A |
| Upregulated genes (alpha=e-15) | *EVI2B* | 2124 | ecotropic viral integration site 2B |
| Upregulated genes (alpha=e-15) | *FAM102A* | 399665 | family with sequence similarity 102 member A |
| Upregulated genes (alpha=e-15) | *FAM3C* | 10447 | FAM3 metabolism regulating signaling molecule C |
| Upregulated genes (alpha=e-15) | *FBXO30* | 84085 | F-box protein 30 |
| Upregulated genes (alpha=e-15) | *FCMR* | 9214 | Fc fragment of IgM receptor |
| Upregulated genes (alpha=e-15) | *FOXL1* | 2300 | forkhead box L1 |
| Upregulated genes (alpha=e-15) | *FOXP1* | 27086 | forkhead box P1 |
| Upregulated genes (alpha=e-15) | *FRAT1* | 10023 | FRAT regulator of WNT signaling pathway 1 |
| Upregulated genes (alpha=e-15) | *GALNT2* | 2590 | polypeptide N-acetylgalactosaminyltransferase 2 |
| Upregulated genes (alpha=e-15) | *GAS7* | 8522 | growth arrest specific 7 |
| Upregulated genes (alpha=e-15) | *GCSAM* | 257144 | germinal center associated signaling and motility |
| Upregulated genes (alpha=e-15) | *GJD3* | 125111 | gap junction protein delta 3 |
| Upregulated genes (alpha=e-15) | *GNG7* | 2788 | G protein subunit gamma 7 |
| Upregulated genes (alpha=e-15) | *HBP1* | 26959 | HMG-box transcription factor 1 |
| Upregulated genes (alpha=e-15) | *HDAC5* | 10014 | histone deacetylase 5 |
| Upregulated genes (alpha=e-15) | *HIPK2* | 28996 | homeodomain interacting protein kinase 2 |
| Upregulated genes (alpha=e-15) | *HOMER2* | 9455 | homer scaffold protein 2 |
| Upregulated genes (alpha=e-15) | *HRK* | 8739 | harakiri, BCL2 interacting protein |
| Upregulated genes (alpha=e-15) | *IL16* | 3603 | interleukin 16 |
| Upregulated genes (alpha=e-15) | *IL4R* | 3566 | interleukin 4 receptor |
| Upregulated genes (alpha=e-15) | *ITGB1* | 3688 | integrin subunit beta 1 |
| Upregulated genes (alpha=e-15) | *ITPRIPL2* | 162073 | ITPRIP like 2 |
| Upregulated genes (alpha=e-15) | *JAK1* | 3716 | Janus kinase 1 |
| Upregulated genes (alpha=e-15) | *KANK2* | 25959 | KN motif and ankyrin repeat domains 2 |
| Upregulated genes (alpha=e-15) | *KLHL14* | 57565 | kelch like family member 14 |
| Upregulated genes (alpha=e-15) | *KLHL24* | 54800 | kelch like family member 24 |
| Upregulated genes (alpha=e-15) | *LAMA5* | 3911 | laminin subunit alpha 5 |
| Upregulated genes (alpha=e-15) | *LAX1* | 54900 | lymphocyte transmembrane adaptor 1 |
| Upregulated genes (alpha=e-15) | *LDLRAD4* | 753 | low density lipoprotein receptor class A domain containing 4 |
| Upregulated genes (alpha=e-15) | *LITAF* | 9516 | lipopolysaccharide induced TNF factor |
| Upregulated genes (alpha=e-15) | *LMNA* | 4000 | lamin A/C |
| Upregulated genes (alpha=e-15) | *LOC112268313* | 112268313 | collagen alpha-1(I) chain-like |
| Upregulated genes (alpha=e-15) | *LY9* | 4063 | lymphocyte antigen 9 |
| Upregulated genes (alpha=e-15) | *MAN1A1* | 4121 | mannosidase alpha class 1A member 1 |
| Upregulated genes (alpha=e-15) | *MAP3K1* | 4214 | mitogen-activated protein kinase kinase kinase 1 |
| Upregulated genes (alpha=e-15) | *MDM4* | 4194 | MDM4 regulator of p53 |
| Upregulated genes (alpha=e-15) | *MME* | 4311 | membrane metalloendopeptidase |
| Upregulated genes (alpha=e-15) | *NATD1* | 256302 | N-acetyltransferase domain containing 1 |
| Upregulated genes (alpha=e-15) | *NCK2* | 8440 | NCK adaptor protein 2 |
| Upregulated genes (alpha=e-15) | *NEIL1* | 79661 | nei like DNA glycosylase 1 |
| Upregulated genes (alpha=e-15) | *NSMCE3* | 56160 | NSE3 homolog, SMC5-SMC6 complex component |
| Upregulated genes (alpha=e-15) | *OGFRL1* | 79627 | opioid growth factor receptor like 1 |
| Upregulated genes (alpha=e-15) | *OXTR* | 5021 | oxytocin receptor |
| Upregulated genes (alpha=e-15) | *P2RY10* | 27334 | P2Y receptor family member 10 |
| Upregulated genes (alpha=e-15) | *PCDH9* | 5101 | protocadherin 9 |
| Upregulated genes (alpha=e-15) | *PHYH* | 5264 | phytanoyl-CoA 2-hydroxylase |
| Upregulated genes (alpha=e-15) | *PIK3CA* | 5290 | phosphatidylinositol-4,5-bisphosphate 3-kinase catalytic subunit alpha |
| Upregulated genes (alpha=e-15) | *PIM1* | 5292 | Pim-1 proto-oncogene, serine/threonine kinase |
| Upregulated genes (alpha=e-15) | *PITPNC1* | 26207 | phosphatidylinositol transfer protein cytoplasmic 1 |
| Upregulated genes (alpha=e-15) | *PLEKHG1* | 57480 | pleckstrin homology and RhoGEF domain containing G1 |
| Upregulated genes (alpha=e-15) | *PLXNC1* | 10154 | plexin C1 |
| Upregulated genes (alpha=e-15) | *PRDM2* | 7799 | PR/SET domain 2 |
| Upregulated genes (alpha=e-15) | *PRKCE* | 5581 | protein kinase C epsilon |
| Upregulated genes (alpha=e-15) | *PYHIN1* | 149628 | pyrin and HIN domain family member 1 |
| Upregulated genes (alpha=e-15) | *RAB43* | 339122 | RAB43, member RAS oncogene family |
| Upregulated genes (alpha=e-15) | *RASGEF1B* | 153020 | RasGEF domain family member 1B |
| Upregulated genes (alpha=e-15) | *RASSF6* | 166824 | Ras association domain family member 6 |
| Upregulated genes (alpha=e-15) | *REPIN1* | 29803 | replication initiator 1 |
| Upregulated genes (alpha=e-15) | *RGS1* | 5996 | regulator of G protein signaling 1 |
| Upregulated genes (alpha=e-15) | *RIPOR1* | 79567 | RHO family interacting cell polarization regulator 1 |
| Upregulated genes (alpha=e-15) | *RMI2* | 116028 | RecQ mediated genome instability 2 |
| Upregulated genes (alpha=e-15) | *RN7SK* | 125050 | RNA component of 7SK nuclear ribonucleoprotein |
| Upregulated genes (alpha=e-15) | *RNA28SN1* | 106632264 | RNA, 28S ribosomal N1 |
| Upregulated genes (alpha=e-15) | *RNA28SN2* | 109864282 | RNA, 28S ribosomal N2 |
| Upregulated genes (alpha=e-15) | *RNA28SN3* | 109910382 | RNA, 28S ribosomal N3 |
| Upregulated genes (alpha=e-15) | *RNA28SN4* | 109864272 | RNA, 28S ribosomal N4 |
| Upregulated genes (alpha=e-15) | *RNA45SN1* | 106631777 | RNA, 45S pre-ribosomal N1 |
| Upregulated genes (alpha=e-15) | *RNA45SN2* | 109864279 | RNA, 45S pre-ribosomal N2 |
| Upregulated genes (alpha=e-15) | *RNA45SN3* | 109910379 | RNA, 45S pre-ribosomal N3 |
| Upregulated genes (alpha=e-15) | *RNA45SN4* | 109864271 | RNA, 45S pre-ribosomal N4 |
| Upregulated genes (alpha=e-15) | *RUBCNL* | 80183 | rubicon like autophagy enhancer |
| Upregulated genes (alpha=e-15) | *SAV1* | 60485 | salvador family WW domain containing protein 1 |
| Upregulated genes (alpha=e-15) | *SCN4A* | 6329 | sodium voltage-gated channel alpha subunit 4 |
| Upregulated genes (alpha=e-15) | *SEMA7A* | 8482 | semaphorin 7A (John Milton Hagen blood group) |
| Upregulated genes (alpha=e-15) | *SLC30A4* | 7782 | solute carrier family 30 member 4 |
| Upregulated genes (alpha=e-15) | *SLC35E3* | 55508 | solute carrier family 35 member E3 |
| Upregulated genes (alpha=e-15) | *SLC44A2* | 57153 | solute carrier family 44 member 2 |
| Upregulated genes (alpha=e-15) | *SLC4A10* | 57282 | solute carrier family 4 member 10 |
| Upregulated genes (alpha=e-15) | *SMAD1* | 4086 | SMAD family member 1 |
| Upregulated genes (alpha=e-15) | *SNTB1* | 6641 | syntrophin beta 1 |
| Upregulated genes (alpha=e-15) | *SNX9* | 51429 | sorting nexin 9 |
| Upregulated genes (alpha=e-15) | *SOCS1* | 8651 | suppressor of cytokine signaling 1 |
| Upregulated genes (alpha=e-15) | *STAT2* | 6773 | signal transducer and activator of transcription 2 |
| Upregulated genes (alpha=e-15) | *SUSD3* | 203328 | sushi domain containing 3 |
| Upregulated genes (alpha=e-15) | *SYT17* | 51760 | synaptotagmin 17 |
| Upregulated genes (alpha=e-15) | *TAGAP* | 117289 | T cell activation RhoGTPase activating protein |
| Upregulated genes (alpha=e-15) | *TCP11L2* | 255394 | t-complex 11 like 2 |
| Upregulated genes (alpha=e-15) | *TMX4* | 56255 | thioredoxin related transmembrane protein 4 |
| Upregulated genes (alpha=e-15) | *TNFSF8* | 944 | TNF superfamily member 8 |
| Upregulated genes (alpha=e-15) | *TP53INP1* | 94241 | tumor protein p53 inducible nuclear protein 1 |
| Upregulated genes (alpha=e-15) | *TP63* | 8626 | tumor protein p63 |
| Upregulated genes (alpha=e-15) | *TSC22D3* | 1831 | TSC22 domain family member 3 |
| Upregulated genes (alpha=e-15) | *VAV3* | 10451 | vav guanine nucleotide exchange factor 3 |
| Upregulated genes (alpha=e-15) | *WNK2* | 65268 | WNK lysine deficient protein kinase 2 |
| Upregulated genes (alpha=e-15) | *YPEL3* | 83719 | yippee like 3 |
| Upregulated genes (alpha=e-15) | *ZDBF2* | 57683 | zinc finger DBF-type containing 2 |
| Upregulated genes (alpha=e-15) | *ZEB2* | 9839 | zinc finger E-box binding homeobox 2 |
| Upregulated genes (alpha=e-15) | *ZNF385B* | 151126 | zinc finger protein 385B |
| Upregulated genes (alpha=e-15) | *ZNF521* | 25925 | zinc finger protein 521 |

**Supplementary Table S6**

| **Previously described MYC signatures that are significantly enriched with downregulated genes following the treatment of 5 nM tafasitamab in SU-DHL-6** | | | | | | |
| --- | --- | --- | --- | --- | --- | --- |
| Signatures DB | Category | signatures name | Signature links | Measured genes | Enrichment score | P value (GSEA) |
| MolSigDBv7_1_d202008 | h: hallmark gene sets | HALLMARK_MYC_TARGETS_V2 | <http://www.gsea-msigdb.org/gsea/msigdb/cards/HALLMARK_MYC_TARGETS_V2> | 58 | 0,684 | 0,001 |
| MolSigDBv7_1_d202008 | c2: curated gene sets | SCHUHMACHER_MYC_TARGETS_UP | <http://www.gsea-msigdb.org/gsea/msigdb/cards/SCHUHMACHER_MYC_TARGETS_UP> | 79 | 0,684 | 0,001 |
| MolSigDBv7_1_d202008 | c2: curated gene sets | COLLER_MYC_TARGETS_UP | <http://www.gsea-msigdb.org/gsea/msigdb/cards/COLLER_MYC_TARGETS_UP> | 24 | 0,746 | 0,001 |
| StaudtSigDB_202003 | Signaling pathway | Myc_overexpression_1.5x_up | PMID 16273092: Bild et al. Nature 439:353 (2006), Supplemental Table 1; Genes upregulated by myc by 1.5 fold. (short StaudtLab signature name: MYCUp-2) | 88 | 0,589 | 0,001 |
| MolSigDBv7_1_d202008 | c2: curated gene sets | SCHLOSSER_MYC_TARGETS_AND_SERUM_RESPONSE_DN | <http://www.gsea-msigdb.org/gsea/msigdb/cards/SCHLOSSER_MYC_TARGETS_AND_SERUM_RESPONSE_DN> | 48 | 0,663 | 0,001 |
| MolSigDBv7_1_d202008 | c2: curated gene sets | SCHLOSSER_MYC_TARGETS_AND_SERUM_RESPONSE_UP | <http://www.gsea-msigdb.org/gsea/msigdb/cards/SCHLOSSER_MYC_TARGETS_AND_SERUM_RESPONSE_UP> | 48 | 0,583 | 0,001 |
| MolSigDBv7_1_d202008 | h: hallmark gene sets | HALLMARK_MYC_TARGETS_V1 | <http://www.gsea-msigdb.org/gsea/msigdb/cards/HALLMARK_MYC_TARGETS_V1> | 199 | 0,561 | 0,001 |
| StaudtSigDB_202003 | Transcription factor target | Myc_RNAi_OCILy3 | PMID 16760443: Dave et al. NEJM 354:2431 (2006), Fig. 1B. (short StaudtLab signature name: MYCUp-1) | 53 | 0,570 | 0,001 |
| MolSigDBv7_1_d202008 | c2: curated gene sets | SCHLOSSER_MYC_AND_SERUM_RESPONSE_SYNERGY | <http://www.gsea-msigdb.org/gsea/msigdb/cards/SCHLOSSER_MYC_AND_SERUM_RESPONSE_SYNERGY> | 33 | 0,573 | 0,001 |
|  |  |  |  |  |  |  |
| **Previously described MYC signatures that are significantly enriched with downregulated genes following the treatment of 5 nM rituximab in SU-DHL-6** | | | | | | |
| Signatures DB | Category | signatures name | Signature links | Measured genes | Enrichment score | P value (GSEA) |
| MolSigDBv7_1_d202008 | c2: curated gene sets | SCHUHMACHER_MYC_TARGETS_UP | <http://www.gsea-msigdb.org/gsea/msigdb/cards/SCHUHMACHER_MYC_TARGETS_UP> | 79 | 0,694 | 0,001 |
| MolSigDBv7_1_d202008 | h: hallmark gene sets | HALLMARK_MYC_TARGETS_V2 | <http://www.gsea-msigdb.org/gsea/msigdb/cards/HALLMARK_MYC_TARGETS_V2> | 58 | 0,767 | 0,001 |
| MolSigDBv7_1_d202008 | c2: curated gene sets | SCHLOSSER_MYC_TARGETS_AND_SERUM_RESPONSE_DN | <http://www.gsea-msigdb.org/gsea/msigdb/cards/SCHLOSSER_MYC_TARGETS_AND_SERUM_RESPONSE_DN> | 48 | 0,768 | 0,001 |
| MolSigDBv7_1_d202008 | c2: curated gene sets | COLLER_MYC_TARGETS_UP | <http://www.gsea-msigdb.org/gsea/msigdb/cards/COLLER_MYC_TARGETS_UP> | 24 | 0,678 | 0,001 |
| MolSigDBv7_1_d202008 | c2: curated gene sets | SCHLOSSER_MYC_TARGETS_AND_SERUM_RESPONSE_UP | <http://www.gsea-msigdb.org/gsea/msigdb/cards/SCHLOSSER_MYC_TARGETS_AND_SERUM_RESPONSE_UP> | 48 | 0,676 | 0,001 |
| StaudtSigDB_202003 | Signaling pathway | Myc_overexpression_1.5x_up | PMID 16273092: Bild et al. Nature 439:353 (2006), Supplemental Table 1; Genes upregulated by myc by 1.5 fold. (short StaudtLab signature name: MYCUp-2) | 88 | 0,596 | 0,001 |
| MolSigDBv7_1_d202008 | c2: curated gene sets | ODONNELL_TARGETS_OF_MYC_AND_TFRC_DN | <http://www.gsea-msigdb.org/gsea/msigdb/cards/ODONNELL_TARGETS_OF_MYC_AND_TFRC_DN> | 46 | 0,575 | 0,001 |
| MolSigDBv7_1_d202008 | c2: curated gene sets | PID_MYC_ACTIV_PATHWAY | <http://www.gsea-msigdb.org/gsea/msigdb/cards/PID_MYC_ACTIV_PATHWAY> | 79 | 0,501 | 0,001 |
| MolSigDBv7_1_d202008 | h: hallmark gene sets | HALLMARK_MYC_TARGETS_V1 | <http://www.gsea-msigdb.org/gsea/msigdb/cards/HALLMARK_MYC_TARGETS_V1> | 199 | 0,617 | 0,001 |
| MolSigDBv7_1_d202008 | c2: curated gene sets | CAIRO_PML_TARGETS_BOUND_BY_MYC_UP | <http://www.gsea-msigdb.org/gsea/msigdb/cards/CAIRO_PML_TARGETS_BOUND_BY_MYC_UP> | 22 | 0,614 | 0,001 |
| StaudtSigDB_202003 | Transcription factor target | Myc_RNAi_OCILy3 | PMID 16760443: Dave et al. NEJM 354:2431 (2006), Fig. 1B. (short StaudtLab signature name: MYCUp-1) | 53 | 0,553 | 0,001 |
| MolSigDBv7_1_d202008 | c2: curated gene sets | DANG_MYC_TARGETS_UP | <http://www.gsea-msigdb.org/gsea/msigdb/cards/DANG_MYC_TARGETS_UP> | 128 | 0,512 | 0,001 |
| MolSigDBv7_1_d202008 | c2: curated gene sets | DANG_REGULATED_BY_MYC_UP | <http://www.gsea-msigdb.org/gsea/msigdb/cards/DANG_REGULATED_BY_MYC_UP> | 68 | 0,585 | 0,001 |
| MolSigDBv7_1_d202008 | c2: curated gene sets | SCHLOSSER_MYC_AND_SERUM_RESPONSE_SYNERGY | <http://www.gsea-msigdb.org/gsea/msigdb/cards/SCHLOSSER_MYC_AND_SERUM_RESPONSE_SYNERGY> | 33 | 0,630 | 0,001 |
| MolSigDBv7_1_d202008 | c2: curated gene sets | SCHLOSSER_MYC_TARGETS_REPRESSED_BY_SERUM | <http://www.gsea-msigdb.org/gsea/msigdb/cards/SCHLOSSER_MYC_TARGETS_REPRESSED_BY_SERUM> | 159 | 0,526 | 0,001 |
| MolSigDBv7_1_d202008 | c2: curated gene sets | YU_MYC_TARGETS_UP | <http://www.gsea-msigdb.org/gsea/msigdb/cards/YU_MYC_TARGETS_UP> | 43 | 0,562 | 0,001 |
| StaudtSigDB_202003 | Transcription factor target | Myc_ChIP_PET_Expr_Up | PMID 17093053: Zeller et al. PNAS 103:17834 (2006), Table ST5. (short StaudtLab signature name: MycUp-4) | 403 | 0,543 | 0,001 |
|  |  |  |  |  |  |  |
| **Previously described MYC signatures that are significantly enriched with downregulated genes following the combination treatment of 5 nM tafasitamab and 5 nM rituximab in SU-DHL-6** | | | | | | |
| Signatures DB | Category | signatures name | Signature links | Measured genes | Enrichment score | P value (GSEA) |
| MolSigDBv7_1_d202008 | h: hallmark gene sets | HALLMARK_MYC_TARGETS_V2 | <http://www.gsea-msigdb.org/gsea/msigdb/cards/HALLMARK_MYC_TARGETS_V2> | 58 | 0,772 | 0,001 |
| MolSigDBv7_1_d202008 | c2: curated gene sets | SCHLOSSER_MYC_TARGETS_AND_SERUM_RESPONSE_DN | <http://www.gsea-msigdb.org/gsea/msigdb/cards/SCHLOSSER_MYC_TARGETS_AND_SERUM_RESPONSE_DN> | 48 | 0,805 | 0,001 |
| MolSigDBv7_1_d202008 | c2: curated gene sets | SCHUHMACHER_MYC_TARGETS_UP | <http://www.gsea-msigdb.org/gsea/msigdb/cards/SCHUHMACHER_MYC_TARGETS_UP> | 79 | 0,729 | 0,001 |
| MolSigDBv7_1_d202008 | c2: curated gene sets | COLLER_MYC_TARGETS_UP | <http://www.gsea-msigdb.org/gsea/msigdb/cards/COLLER_MYC_TARGETS_UP> | 24 | 0,738 | 0,001 |
| StaudtSigDB_202003 | Signaling pathway | Myc_overexpression_1.5x_up | PMID 16273092: Bild et al. Nature 439:353 (2006), Supplemental Table 1; Genes upregulated by myc by 1.5 fold. (short StaudtLab signature name: MYCUp-2) | 88 | 0,636 | 0,001 |
| MolSigDBv7_1_d202008 | c2: curated gene sets | SCHLOSSER_MYC_TARGETS_AND_SERUM_RESPONSE_UP | <http://www.gsea-msigdb.org/gsea/msigdb/cards/SCHLOSSER_MYC_TARGETS_AND_SERUM_RESPONSE_UP> | 48 | 0,777 | 0,001 |
| StaudtSigDB_202003 | Transcription factor target | Myc_RNAi_OCILy3 | PMID 16760443: Dave et al. NEJM 354:2431 (2006), Fig. 1B. (short StaudtLab signature name: MYCUp-1) | 53 | 0,564 | 0,001 |
| MolSigDBv7_1_d202008 | c2: curated gene sets | CAIRO_PML_TARGETS_BOUND_BY_MYC_UP | <http://www.gsea-msigdb.org/gsea/msigdb/cards/CAIRO_PML_TARGETS_BOUND_BY_MYC_UP> | 22 | 0,581 | 0,001 |
| MolSigDBv7_1_d202008 | c2: curated gene sets | DANG_REGULATED_BY_MYC_UP | <http://www.gsea-msigdb.org/gsea/msigdb/cards/DANG_REGULATED_BY_MYC_UP> | 68 | 0,571 | 0,001 |
| MolSigDBv7_1_d202008 | h: hallmark gene sets | HALLMARK_MYC_TARGETS_V1 | <http://www.gsea-msigdb.org/gsea/msigdb/cards/HALLMARK_MYC_TARGETS_V1> | 199 | 0,640 | 0,001 |
| MolSigDBv7_1_d202008 | c2: curated gene sets | SCHLOSSER_MYC_TARGETS_REPRESSED_BY_SERUM | <http://www.gsea-msigdb.org/gsea/msigdb/cards/SCHLOSSER_MYC_TARGETS_REPRESSED_BY_SERUM> | 159 | 0,535 | 0,001 |
| StaudtSigDB_202003 | Transcription factor target | Myc_ChIP_PET_Expr_Up | PMID 17093053: Zeller et al. PNAS 103:17834 (2006), Table ST5. (short StaudtLab signature name: MycUp-4) | 403 | 0,563 | 0,001 |
| MolSigDBv7_1_d202008 | c2: curated gene sets | SCHLOSSER_MYC_AND_SERUM_RESPONSE_SYNERGY | <http://www.gsea-msigdb.org/gsea/msigdb/cards/SCHLOSSER_MYC_AND_SERUM_RESPONSE_SYNERGY> | 33 | 0,664 | 0,001 |
| MolSigDBv7_1_d202008 | c2: curated gene sets | ACOSTA_PROLIFERATION_INDEPENDENT_MYC_TARGETS_UP | <http://www.gsea-msigdb.org/gsea/msigdb/cards/ACOSTA_PROLIFERATION_INDEPENDENT_MYC_TARGETS_UP> | 83 | 0,514 | 0,001 |

**Supplementary Table S7**

| **Synergy analysis for mice survival by applying Cox proportional hazards model** | | | |
| --- | --- | --- | --- |
| Combination | Variable | Hazard ratio | P value |
| Tafasitamab 0,3 mg/kg + rituximab 0,3 mg/kg | Tafasitamab | 0,539 | 0,2 |
|  | Rituximab | 0,447 | 0,12 |
|  | PBMCs | 0,216 | 0,004 |
|  | Tafasitamab:rituximab | 0,559 | 0,41 |
| Tafasitamab 1 mg/kg + rituximab 0,3 mg/kg | Tafasitamab | 0,099 | 2,40E-04 |
|  | Rituximab | 0,296 | 0,036 |
|  | PBMCs | 0,258 | 0,012 |
|  | Tafasitamab:rituximab | 2,004 | 0,39 |
| Tafasitamab 1 mg/kg + rituximab 0,6 mg/kg | Tafasitamab | 0,028 | 5,90E-05 |
|  | Rituximab | 0,017 | 1,60E-05 |
|  | PBMCs | 0,192 | 0,009 |
|  | Tafasitamab:rituximab | 12,068 | 0,042 |
